# Ancillary health effects of climate mitigation scenarios as drivers of policy uptake: a review of air quality, transportation and diet co-benefits modeling studies

**DOI:** 10.1088/1748-9326/aa8f7b

**Published:** 2017-10-27

**Authors:** Kelly M Chang, Jeremy J Hess, John M Balbus, Jonathan J Buonocore, David A Cleveland, Maggie L Grabow, Roni Neff, Rebecca K Saari, Christopher W Tessum, Paul Wilkinson, Alistair Woodward, Kristie L Ebi

**Affiliations:** 1 University of Washington Center for Health and the Global Environment, Seattle, WA 98105, United States of America; 2 National Institute of Environmental Health Sciences, Durham, NC, United States of America; 3 Center for Health and the Global Environment, Harvard School of Public Health, Landmark Center 4th Floor, Suite 415, 401 Park Drive, Boston, MA 02215, United States of America; 4 University of California Santa Barbara, Santa Barbara, CA, United States of America; 5 Family Medicine and Community Health, University of Wisconsin Madison School of Medicine and Public Health, 1100 Delaplaine Ct, Madison, WI 53715, United States of America; 6 Johns Hopkins University Bloomberg School of Public Health, Baltimore, MD, United States of America; 7 University of Waterloo, Waterloo, Ontario, Canada; 8 University of Washington, Seattle, WA, United States of America; 9 London School of Hygiene and Tropical Medicine, University of London, London, United Kingdom; 10 University of Auckland, Auckland, New Zealand; 11 LLC, ClimAdapt, 424 Tyndall Street, Los Altos, CA 94022, United States of America

**Keywords:** greenhouse gases, health co-benefits, climate mitigation, modeling, diet, air quality, transportation

## Abstract

**Background:**

Significant mitigation efforts beyond the Nationally Determined Commitments (NDCs) coming out of the 2015 Paris Climate Agreement are required to avoid warming of 2°C above pre-industrial temperatures. Health co-benefits represent selected near term, positive consequences of climate policies that can offset mitigation costs in the short term before the beneficial impacts of those policies on the magnitude of climate change are evident. The diversity of approaches to modeling mitigation options and their health effects inhibits meta-analyses and syntheses of results useful in policy-making.

**Methods/Design:**

We evaluated the range of methods and choices in modeling health co-benefits of climate mitigation to identify opportunities for increased consistency and collaboration that could better inform policy-making. We reviewed studies quantifying the health co-benefits of climate change mitigation related to air quality, transportation, and diet published since the 2009 Lancet Commission ‘Managing the health effects of climate change’ through January 2017. We documented approaches, methods, scenarios, health-related exposures, and health outcomes.

**Results/Synthesis:**

Forty-two studies met the inclusion criteria. Air quality, transportation, and diet scenarios ranged from specific policy proposals to hypothetical scenarios, and from global recommendations to stakeholder-informed local guidance. Geographic and temporal scope as well as validity of scenarios determined policy relevance. More recent studies tended to use more sophisticated methods to address complexity in the relevant policy system.

**Discussion:**

Most studies indicated significant, nearer term, local ancillary health benefits providing impetus for policy uptake and net cost savings. However, studies were more suited to describing the interaction of climate policy and health and the magnitude of potential outcomes than to providing specific accurate estimates of health co-benefits. Modeling the health co-benefits of climate policy provides policy-relevant information when the scenarios are reasonable, relevant, and thorough, and the model adequately addresses complexity. Greater consistency in selected modeling choices across the health co-benefits of climate mitigation research would facilitate evaluation of mitigation options particularly as they apply to the NDCs and promote policy uptake.

## Introduction

The Nationally Determined Contributions (NDCs) underlying the2015 Paris Agreement under the United Nations Framework Convention on Climate Change (UNFCCC) committed countries to deep reductions in global greenhouse gas (GHG) emissions to hold the global average temperature below a 2 °C increase above pre-industrial levels. The agreement is an important step forward, but by 2030 significant emissions reductions beyond the current NDCs will be required to limit warming within the 2 °C threshold (Fujimori et al 2016), prompting countries to consider the range of climate policy options and their broader impacts.

**Figure 1.**
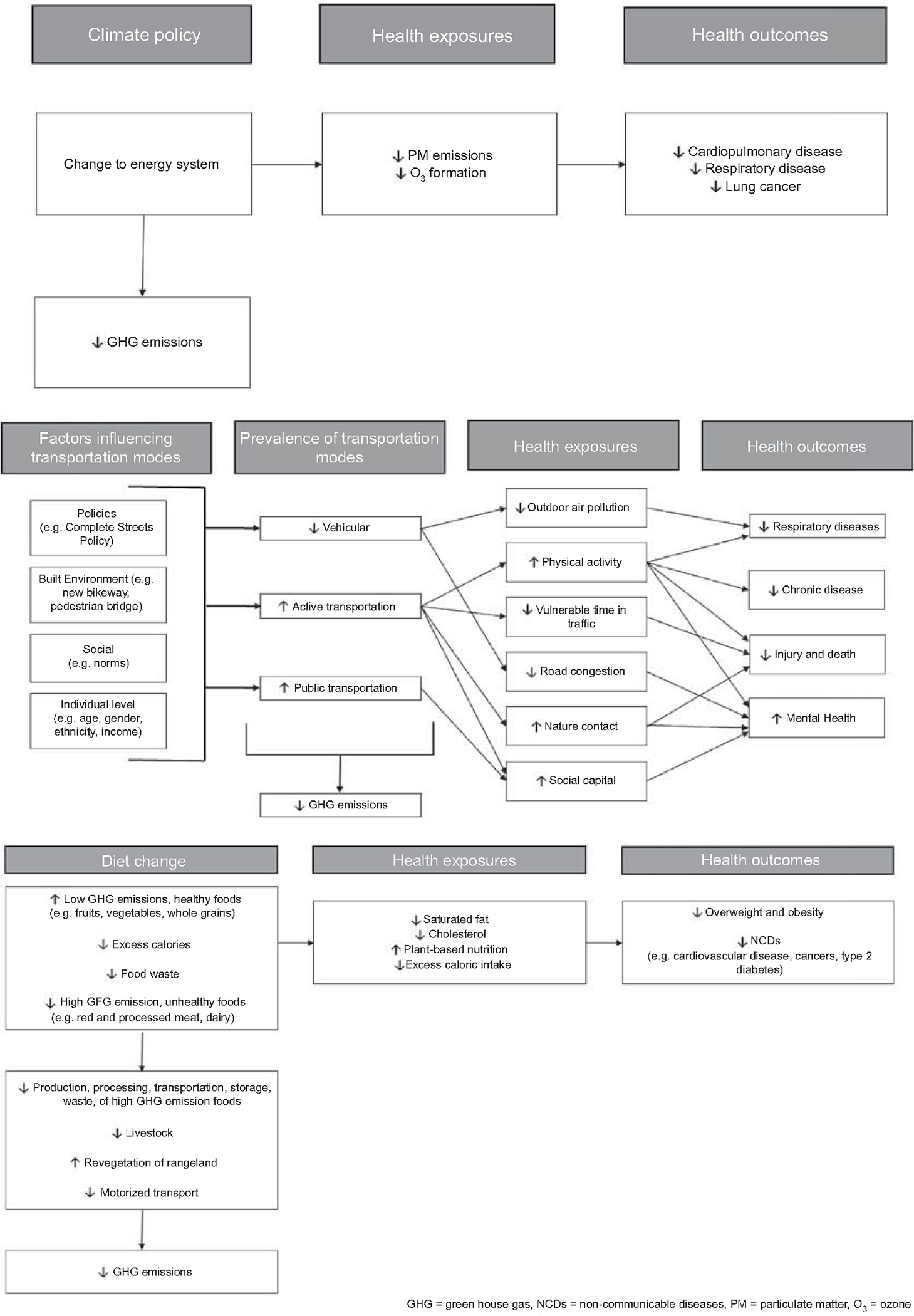
(*a*) Conceptual framework linking climate mitigation and health outcomes via air quality. (*b*) Conceptual framework linking climate mitigation and health outcomes via transportation. (*c*) Conceptual framework linking climate mitigation and health outcomes via diet.

The cost of implementing mitigation policies that could achieve GHG emissions reductions agreed to in the Paris Agreement is estimated to be several percent of global gross domestic product by mid-century (Boyd et al 2015). However, economic assessments rarely include associated health co-benefits even though mitigation policies and technologies influence health by modifying health-related exposures such as non-GHG air pollutants, physical activity, and diet. Conceptual frameworks demonstrating links between air quality, transportation, and diet-related climate mitigation activities and health exposures and outcomes are presented in figure 1. Ignoring cost savings due to health impacts provides an unbalanced assessment of the net impacts of required mitigation activities.

The health co-benefits literature has expanded significantly since publication of *The Lancet* series of papers linking climate mitigation and health in November 2009 (Haines et al 2009). *The Lancet* papers provided a quantitative and methodological foundation for evaluating the costs and health co-benefits of mitigation policies and activities. Since then, two papers (Hosking and Campbell-Lendrum 2012, Smith et al 2016) reviewed the literature on the ancillary effects of mitigation activities on health outcomes, and a third (Nemet et al 2010) reviewed valuations of air quality co-benefits of mitigation and their relevance to policy cost-benefit analysis, but did not discuss quantification of health outcomes specifically. Hosking and Campbell-Lendrum (2012) produced a scoping review evaluating the match between the needs of policy-makers and the available research relating to climate change and quantification of health-specific outcomes. Their review was limited to research published as of June 2010 and since the World Health Assembly (WHA) established five research priority areas related to climate change-related health threats in 2008 (WHA2008). Although climate change and health studies nearly doubled in the two years investigated, the authors identified only 12 studies of co-benefits and co-harms associated with climate mitigation and a dearth of studies pertaining to co-benefits in developing regions. Smith et al (2016) conducted a systematic, semi-quantitative review applying published estimates of health co-benefits to quantify the relative magnitude of health and environmental effects related to implementation of the UK Committee on Climate Change (CCC) 2008 to 2027 carbon budget (Smith et al 2016).

For co-benefits studies to support a case for or against a particular climate policy, Jack and Kinney (2010) suggest they must specify: meaningful scenarios, translation of policy into behavior, influence of behavior on emissions, relationship of emissions to health-determinant exposures, and quantification of health outcomes as a result of exposure. In the authors’ words, ‘the policy impact of the co-benefits literature will be proportional to its ability to link credible models of economic behavior, environmental processes, and health’ (Jack and Kinney 2010). Noting the persistent diversity in modeling choices among the health co-benefits of mitigation studies, Remais et al (2014) also recommended increased rigor in the treatment of uncertainty and discount rates, inclusion of the range of ancillary health impacts (i.e. positive and negative effects), collaboration with policy makers in analytical choices, and consideration of low-probability, high-impact events such as nuclear accidents. Most recently, Liu et al (2017)suggested that in order for a model to provide policy-relevant science it should (1) be universal so the outputs are comparable, (2) facilitate rapid-calculation for simulating multiple scenarios, and (3) utilize input data that is accessible and straightforward.

We review studies published over the last eight years modeling the health co-benefits of mitigation policies and activities related to air quality, transportation, and diet. Our aim is to document the diversity of approaches, modeling methods, policy scenarios, assumptions, and time slices of the collected studies so that they may be considered with respect to their utility for policy making and evaluation. Climate mitigation policy may involve a range of strategies and interventions in many sectors such as building, industry, infrastructure, and agriculture, but to achieve a manageable scope while still representing the diversity of modeling choices and approaches, we limited this review to air quality, transportation, and diet, for which there is substantial health co-benefits modeling in the literature. We also identify research areas requiring consistency to inform policy decisions and promote policy uptake.

## Methods

We conducted a comprehensive review of quantitative estimates of health co-benefits of climate change mitigation policies in the areas of air quality, transportation, and diet initiatives starting with and published since *The Lancet* series, specifically November 2009 through January 2017. We searched PubMed, Medline, Embase, and Web of Science using the search terms ‘health co-benefits’ and ‘climate mitigation’ and synonyms for each sector (see supplementary table 1 available at stacks.iop.org/ERL/12/113001/mmedia for a complete list of search terms). Because our intent was to be comprehensive, the focus was on identifying all potentially relevant literature. For example, within the timeframe of interest in PubMed, the 11 combinations of search terms for air quality identified 496 potential citations; the 17 combinations of search terms for transportation identified 2818 potential citations; and the 8 combinations of search terms for diet identified 99 potential citations. Larger numbers of potential citations were identified using Embase and Web of Science, with large overlap. We also reviewed citations in articles uncovered in our searches and included publications brought to our attention by co-benefits researchers.

The inclusion criteria were that the abstract indicated the study was a modeling study that (1) quantified population level health outcomes, (2) related to changes in exposure(s), and (3) correlated with a specified climate mitigation scenario or policy. Studies meeting these criteria and focusing on the primary sectors of interest were included for full review. A standardized information capture matrix (refer to supplementary tables 2, 3 and 4) was developed *a priori* and used by the reviewers (KMC, KLE, JJH, RdB, RKS, MLG, and DAC). Studies were compared with regards to scenario construction, policy relevance, baseline, health-related exposures, health outcomes, geographic and temporal scale, and, when reported, health co-benefit valuation and proportional emissions reduction.

Some studies estimated health endpoints as a means toward monetizing co-benefits but did not explicitly describe their modeling or report health outcomes (e.g. Buonocore et al 2016, Siler-Evans et al 2013). While mortality could be back calculated using an estimate of the value of a statistical life, these studies did not meet our inclusion criteria because they did not report a quantification of a health outcome. However, studies that calculated mortality estimates from valuations themselves, such as studies employing the Health Economic Assessment Tool for walking and biking, which outputs monetary savings (e.g. Creutzig et al 2012), were included because they quantify and report a health outcome as an intermediate step. Studies that calculated impacts on the Canadian Air Quality Health Index (AQHI) (e.g. Kelly et al 2012) were also excluded because AQHI is not a specific health outcome.

Rebound, defined as when the savings (either in health outcomes or GHG emissions reductions) are reinvested in other activities that generate GHG emissions or disease, has the potential to negate some or all of the savings from mitigation efforts (Font Vivanco et al 2016). Rebound has important policy implications, but is not within the scope of this review.

## Results

Forty-two studies published from November 2009 through January 2017 met the inclusion criteria and quantified health co-benefits of climate mitigation, including 24 addressing air quality exposures (supplementary table 2), 12 estimating exposures related to transportation such as physical activity (supplementary table 3), and six that modeled diet-related exposures (supplementary table 4).

Overall, studies quantifying the health co-benefits of climate mitigation efforts adhered to the scoping framework outlined by Remais et al (2014) and specified: mitigation strategy, association with health drivers, population, time scale, and baseline trends in demographics, health-related exposures, and health, and finally, health impact assessment (i.e. change in health driver and health outcome). Most, but not all, studies conducted sensitivity or uncertainty analysis. Just over half reported the health co-benefits in monetary terms in addition to specific health outcomes. Studies utilized a range of population-specific data sources where available and in many cases employed standard sector-specific economic, atmospheric, transportation, health impact, and climate models. Studies relied on epidemiological literature to specify concentration response functions describing the relationship between exposure and health outcome, often stratified by relevant population segments. In most instances, studies had to rely on epidemiological studies derived from populations other than the study population.

**Figure 2.**
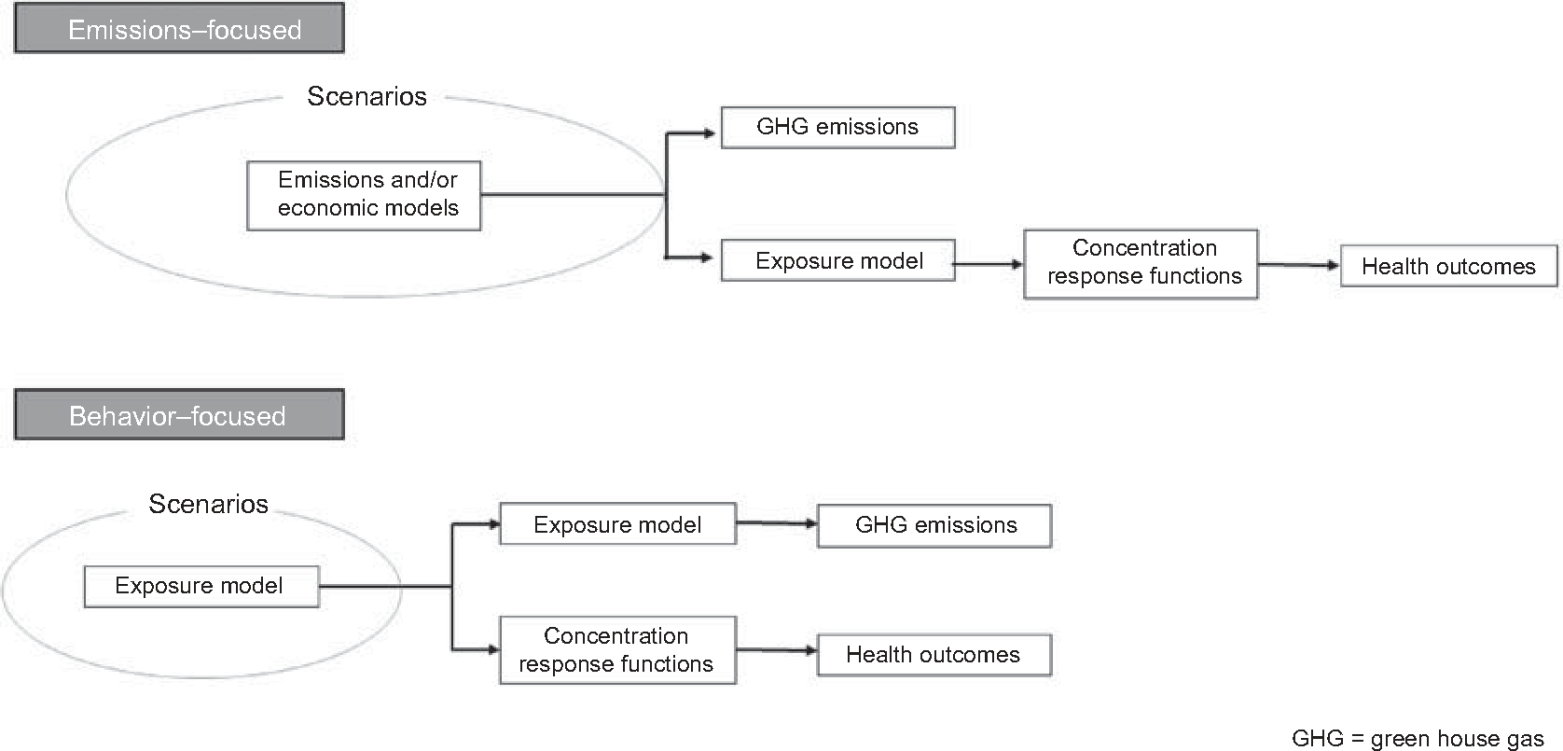
Two approaches to defining modeling scenarios in health co-benefits of climate mitigation studies

Studies took one of two approaches to defining the modeled scenario: emissions-focused or behavior-focused (figure 2). An emissions-focused approach, typical of but not exclusive to air quality co-benefits studies, investigated the health outcomes associated with mitigation scenarios that impact GHG emissions and have a secondary but simultaneous effect on health-related exposures such as air pollutants. Behavior-focused studies considered a change in a behavior at the population level, such as a reduction in motor vehicle transport or reduced consumption of meat, that impacts both health-determining exposures and GHG emissions. The behavior-focused approach was typical of transportation and diet health co-benefits studies.

Theoretical frameworks were used to elaborate the pathways between an intervention and its climate and health effects, and could be used to specify which pathways were and were not included in the scope of the analysis (e.g. Liu et al 2017, Xia et al 2015, Woodcock et al 2009). Causal loop diagrams were used in one case to illustrate positive and negative feedbacks and complexity in the modeled system (Macmillan et al 2014).

### Air quality

Combatting climate change can reduce air pollution through two main mechanisms: (1) directly, by reducing the climate penalty on air quality (described below), and (2) indirectly, by reducing co-emitted air pollutants. In the US, the latter mechanism will have the greatest impact on air pollution and therefore on health at least until mid-century (Garcia-Menendez et al 2015). Power plants, certain industrial processes, mobile sources, and agricultural activities are sources of GHG emissions, including carbon (CO_2_) and methane, that contribute to anthropogenic climate change (IPCC 2014). At the same time, these sources can emit a range of pollutant particles and gases that directly or indirectly affect health and increase the risk of premature deaths (Burnett et al 2014, Lepeule et al 2012, Krewski et al 2009). Ambient air pollution from particulate matter (PM) and ozone (O_3_) was estimated to cause nearly 4.5 million deaths worldwide in 2015 (Cohen et al 2017). Therefore, reducing emissions from these sources could contribute to reducing GHG emissions and would benefit public health. Further, higher temperatures associated with climate change may increase health risks by increasing the secondary formation of PMandO_3_,aphenomenondubbedthe’climatechange penalty’ on air pollution (Silva et al 2013, Wu et al 2008, Fiore et al 2015).

There is a growing literature estimating the health co-benefits of reducing co-emitted pollutants and the climate change penalty. The 24 included studies take a diversity of approaches in estimating health co-benefits in terms of geographic scale, specificity of the scenario analyzed, policy relevance, pollutant exposures, health outcomes, valuation, and other factors (supplementary table 2). Although all report health co-benefits from the mitigation policies investigated, the range of approaches makes it difficult to synthesize beyond general statements.

#### Approaches

Motivations for the studies ranged from estimating current co-benefits of a specific policy proposed for a city or country to estimating future co-benefits globally under different mitigation scenarios. Therefore, the geographic and temporal scales differed. The choices of geographic and temporal scale further influenced the specificity of the scenario analyzed, with more detailed scenarios generally assessed at smaller scales.

The level of detail in each step in the analytic chain (i.e. estimating emissions related to a mitigation policy or scenario, modeling resultant changes in air quality, estimating the health impacts based on concentration-response functions) varied, making synthesis of studies challenging. Some studies started with detailed models developed to generate insights into the costs of mitigation policies (e.g. Rao et al 2013); the resultant changes in air pollutants were then coupled with a limited number of health concentration-response functions to estimate co-benefits. These studies had more depth in exploring mitigation options but less in exploring the wide range of possible health co-benefits, and thus may underestimate the extent of health co-benefits. Other studies started with detailed models of how a range of health outcomes can be affected by exposure to air pollutants. For example, West et al 2013 focused on several causes of premature mortality. These models were generally developed for other purposes, such as estimating the costs and benefits of air pollution regulation (e.g. BenMAP). A limited mitigation scenario was coupled with these models to estimate co-benefits. These studies explored a narrower range of mitigation options, but provided a more detailed assessment of health co-benefits.

#### Policy scenarios

Studies examined policies relevant at local, national, or international scales. The specificity of some policy scenarios, while useful for examining specific policy choices, restricted their generalizability to other technologies or contexts. For example, Gilmore et al (2010) examined a 500 MW sodium–sulfur battery charged during off-peak times of the day and discharged during peak times to replace four hours of electricity generation from two types of electricity generating peaking plants in New York, US. Eight studies focused on national level policies aimed at overall reductions in CO_2_ to achieve national mitigation goals, energy efficiency measures, improvements in electricity generation, *inter alia*; for example comparing the US Clean Energy Standard with cap and trade policies (Saari et al 2015, Thompson et al 2014). Other studies focused on informing international negotiations on mitigation policies. One such study estimated the health co-benefits in the US of implementing a global carbon tax to achieve radiative forcing levels of 3.7 or 4.5 W m^−2^ in 2100 (Garcia-Menendez et al 2015). Rao et al (2013) compared multiple possible air pollution, energy access, and climate policies to identify which would be associated with the largest health co-benefits.

#### Policy baselines

Baselines to the policy scenario are important for co-benefit estimates because they determine background air quality and the level of additional achievable reductions via counterfactuals. Several near-term studies used current legislation (or a scenario of legislation) to ensure comparability in the baseline and estimated health co-benefit (e.g. West et al 2012). RCP 8.5 (Representative Concentration Pathway of GHG emissions that results in 8.5 W m^−2^ in 2100, approximately the current emissions pathway), for example, was used as the baseline for several studies to represent future conditions under a no mitigation policy scenario (e.g. Schucht et al 2015). There are limited differences in global mean surface temperature across the RCPs until 2050, so RCP 8.5 is often used as a baseline for projections later in the century.

#### Temporal scales

Temporal scales in the study sample ranged widely from present to 2100. The purpose of the study dictated the period of interest. The 2030s and 2050swere frequently used in projections of the magnitude and pattern of the risks of climate change (IPCC 2014), although longer simulations are typically needed to assess climate stabilization.

#### Sources of GHG emissions

Studies varied in the sources and species of GHG covered by the policy scenario. Sources ranged from the full economy to fossil-powered electricity, buildings, agriculture, and transportation. As mentioned, these sources were targeted through specific policies or more general scenarios. Balbus et al (2014) took a different approach, analyzing ten options in the transportation, buildings, and power plant sectors that would account for one US ‘wedge’ of GHG reductions amounting to 19 GtCO_2_ cumulative reduction over 50 years. Three studies focused on sources of methane and black carbon (Sarofim et al 2017, Anenberg et al 2012, West et al 2012), while others focused on CO_2_ or CO_2_e.

#### Modeling considerations

Studies varied in their modeling approaches including their level of sophistication at different points in the pathway from policy to air pollution. Some use detailed analyses to estimate the effect of policies on emissions (e.g. Crawford-Brown et al 2013), and emissions on concentrations (e.g. Shindell et al 2012). The effects of policies on emissions were estimated by selecting blanket reductions (e.g. Markandya et al 2009), using engineering calculations (e.g. Balbus et al 2014), or employing scenarios developed with models of electricity, transportation, or economic systems (e.g. Anenberg et al 2012), or a combination thereof, e.g., within an integrated assessment model (e.g. Rao et al 2016). Atmospheric response to emissions was estimated by chemical transport models to estimate co-benefits from co-emissions, or coupled with climate inputs or feedbacks to capture direct benefits from reducing the climate change penalty. Such methodological variety is often deliberate and appropriate for a specific policy application. However, it introduces an additional difficulty when attempting to draw comparisons across studies by adding variability related to model choice in addition to other sources of variability.

#### Concentration-response function considerations

The studies considered a range of adverse health outcomes, including premature mortality from cardio-respiratory diseases, lung cancer, and acute respiratory infections. Studies of morbidity estimates included hospital admissions, long-term health care, asthma admissions, and restricted activity days.

When no-effects thresholds were applied, they ranged from 7.5–50 *μ*g m^−3^ for PM_2.5_, although there is evidence for risk below this range (Lepeule et al 2012). Thresholds were typically derived from studies conducted in high-income countries, so there are questions regarding the appropriateness of applying the same functions and thresholds in low- and middle-income countries, where concentrations of air pollutants can be much higher. Further, some studies assumed concentration-response relationships were linear and others used non-linear functions (e.g. Rao et al 2013). Additionally, the health benefits of reductions in ambient air pollution can be difficult to model without knowing the background contribution of household air pollution or secondhand smoke to total exposures (Burnett et al 2014).

#### Results

Health co-benefits were reported as reductions in disability adjusted life years (DALYs), years of life lost (YLL), and mortality. The broad range of policy scenarios limits more detailed statements than mitigation policies would result in health co-benefits, with the extent of co-benefits varying by policy specifics, air pollutants considered, and analytic choices of geographic and temporal scale, demographic and socioeconomic changes over the study period, and health outcomes included.

Fourteen of the included studies then monetized the estimated health co-benefits to estimate the extent to which these benefits could offset the costs of implementing the policy. Although all showed some degree of offset of the policies assessed, the differences across the studies in how this was calculated makes general statements challenging.

Nonetheless, studies that compared the effects of co-emitted pollutants to that of the climate change penalty suggest that the first is the most significant, at least by mid-century, for ozone (Selin et al 2009) and fine particulate matter (Garcia-Menendez et al 2015). In addition, several studies reported an estimate of the dollars of air quality co-benefits per ton of CO_2_ avoided. The range across these studies was $2–380 ton^−1^ of CO_2_ avoided, with a maximum nearly double that of earlier reviews yielding $2–196/ton CO_2_ (Nemet et al 2010), and ranges even higher to $700–5000 ton^−1^ for CH4 (Sarofim et al 2017, Shindell et al 2012).

#### Relevance and inclusion of co-harms

While these studies presented overall air quality benefits, several indicated the potential for dis-benefits of climate policy. For example, increased health risks occurred in localized areas due to NO_x_ titration of ozone (Thompson et al 2014). In addition, changes in health-related exposure due to regulation or lack of regulation are not constrained to that area, and ‘leakage’ of health risks was observed in specific unregulated regions or sectors under specific policies (Thompson et al 2016). Thus, distributional considerations could identify dis-benefits for certain stakeholders even if a policy yielded overall health co-benefits.

### Transportation

Twelve studies modeling health outcomes related to emissions reductions scenarios targeting the transportation sector met our inclusion criteria (supplementary table 3). While transportation studies can also include air quality, they included multiple other health impacts and focused on a sector that generates a significant fraction of GHG emissions. In the United States, transportation is the second largest contributor to GHG emissions, accounting for 26% of emissions by economic sector in 2014 (USEPA 2015). The proportion of emissions accounted for by transportation increases as more renewable energy is used in other sectors. For example, transportation contributes 36% of the GHG emissions in the US state of California, the largest emission of any sector (California Environmental Protection Agency Air Resources Board 2016). In New Zealand, where over 80% of electricity is generated by hydropower and geothermal and wind sources, road transport is responsible for 40% of energy emissions (Ministry for the Environment 2017). Therefore, reducing transportation emissions will be important for achieving global, national, and local targets.

Transportation choices affect health in many ways. Car use, for example, increases risk of exposure to: traffic-related injury, physical inactivity, air pollution, and noise, among others (Nieuwenhuijsen and Khreis 2016). Traffic emissions may cause 185 000–330000 annual premature deaths globally (Nieuwenhuijsen and Khreis 2016). However, the casualties of transportation related inactivity outweigh those due to air pollution. In New Zealand, for example, it is estimated that shifting 5% of vehicle kilometers to cycling would avoid about 116 deaths a year due to increased physical activity, and there would be 5–6 fewer deaths a year caused by pollution from vehicle emissions (Lindsay et al 2011). Conditions that are linked to transportation include cardiovascular disease, diabetes, mental illness, some cancers, and obesity, and among children: low birth weight, reduced cognitive function, respiratory infection, and decreased lung function (Nieuwenhuijsen and Khreis 2016). Therefore, transportation policies offer powerful opportunities to reduce related morbidity and mortality and cut GHG emissions at the same time.

#### Approaches

Using behavior-focused approaches, most transportation studies developed hypothetical scenarios involving replacement of a proportion of personal automobile use with walking, cycling, and use of public transportation (Stevenson et al 2016, Xia et al 2015, Macmillan et al 2014, Woodcock et al 2013, Rojas-Rueda et al 2011, Grabow et al 2012, Lindsay et al 2011). Three studies started from an emissions-focused premise evaluating the required shifts in transportation modes (e.g. Creutzig et al 2012, Woodcock et al 2009, Shindell et al 2016). The remaining two studies evaluated both types of scenarios (e.g. Sabel et al 2016, Maizlish et al 2013). Research design increased in sophistication over time, starting from simple models based on assumptions of policy or behavior implementation and general distribution of benefits, to more sophisticated approaches including stratified designs that acknowledged the age-dependency of co-benefits and co-harms (e.g. Woodcock et al 2013).

#### Policy scenarios

Not all included studies had a policy assessment; however, many of them had policy implications and/or laid the groundwork for future policy analyses. For instance, some of the studies could provide support for the enactment of Complete Streets policies, establishment of a bikeshare program, or introduction of congestion charges and other price interventions to reduce use of motor vehicles. Complete Streets policies support active transportation through the routine design, maintenance, and operation of streets and communities that are safe and accommodating for all people, regardless of age, ability, or mode of transport (Carlson et al 2017). On the other hand, some of the scenarios were policy packages developed with stakeholder engagement (e.g. Creutzig et al 2012, Woodcock et al 2009 and 2013). Shindell et al (2016) estimated the climate and health benefits of reducing US emissions consistent with a 2°C increase in global mean surface temperature; these analyses assumed transportation reductions avoiding 0.03 °C warming in 2030 and 0.15 °C in 2100.

#### Policy baselines

Study settings varied widely, from cities to regions to national assessments, and there was little commonality in the interventions themselves, which consisted of various combinations of walking, cycling, and taking public transport. The baseline against which policy scenarios were measured was ‘business as usual’ (e.g. Macmillan et al 2014, Sabel et al 2016).

#### Temporal scales

Studies focused on the health benefits of increasing physical activity by replacing a portion of car trips with active transportation either today or within the next few decades. One study (Shindell et al 2016) projected health co-benefits to the end of the century, but the majority presented results for a decade in the first half of the century. The baselines were generally present day or a recent period.

#### Sources of GHG emissions

Burning fossil fuels is the primary source of transportation emissions; over 90% of the fuel used for transportation is petroleum-based, including gasoline and diesel. Studies reviewed included operational GHG emissions from motor vehicle road traffic, in particular commuter motor vehicles.

#### Modeling considerations

Over time, studies employed increasingly sophisticated approaches to estimate the co-benefits and co-harms of increasing active transport and considered the benefits of increased physical activity alongside the risks of injury and road traffic fatalities, and exposure to air pollution, noise, well-being, and other factors. Increasing method sophistication included use of age, gender, and fitness stratification (Woodcock et al 2013), consideration of social and cultural factors (e.g. the extent to which biking is considered normal or even favored), and inclusion of infrastructure parameters (e.g. the extent and safety of bike lanes). More recent studies used system dynamics modeling to address the complexity inherent to transportation policy including system feedbacks, interacting variables as drivers of system behavior, and time-dependency of cause-and-effect relationships that may produce trade-offs between long-term and short-term policy effects (Macmillan et al 2014).

#### Concentration- and exposure-response function considerations

The studies relied on quantifications of the adverse health consequences of exposure to air pollution. The studies analyzed cardio-respiratory diseases, other chronic diseases, road traffic fatalities, obesity, well-being (e.g. mental health), and other health outcomes, with mortality the most frequent endpoint considered. The studies were conducted predominantly in high income countries; it is uncertain the extent to which they would be applicable to low- and middle-income countries where infrastructure, density of settlement, traffic conditions, and vehicle speeds vary greatly. Stevenson et al (2016) is an example of a transportation modeling study that investigated co-benefits in a range of lower and higher income countries.

Transportation health co-benefits modeling studies focused on classic health outcomes such as cardiovascular disease, overweight, and all-cause mortality. Other important health-related effects of reduction in motor vehicle transport may be included in future studies. For instance, with one exception (Maizlish et al 2013), these models have not yet incorporated social-emotional well-being and mental health outcomes affected by social severance (e.g. diminished social interactions in neighborhoods divided by roads with high volumes of motor traffic).

#### Results

Significant reductions in DALYs, YLL, and/or mortality were reported from active transport even when potential injuries were considered (see supplementary table 3). A more detailed synthesis is not possible because of differences across the studies in baselines and time slices. Detailed longitudinal assessments, such as those incorporated in the ITHIM tool (Wood cock et al 2013), are needed to fully capture the cumulative benefits of increased physical activity.

Lindsay et al (2011), Macmillan et al (2014), Grabow et al (2012), and Shindell et al (2016) estimated the economic benefits. Lindsay et al (2011) estimated a savings of over NZ$1 million per 1000 commuter cyclists per year in New Zealand, and Shindell et al (2016) estimated near-term benefits of about US$250 billion annually in the US for implementing ambitious policies promoting clean energy and vehicles, depending on assumptions and the discount rate used; these benefits would likely exceed implementation costs. Grabow et al (2012) also estimated economic benefits of $8.7 billion annually over the course of the months when it would be the most feasible for active transportation in the 11 largest cities in the Upper Midwest of the US. Macmillan et al (2014) provided a detailed cost-benefit analysis incorporating implementation costs of commuter cycling policies and their health-related savings due to reduced mortality, hospitalizations, and disease incidence.

#### Relevance and inclusion of co-harms

One concern of increased active transport is potential exposure to cycle-car and pedestrian-car crashes. Injuries and crashes were considered along dimensions of striking vehicle mode, exposure per distance traveled, vehicle or non-vehicle occupant type (e.g. cyclist, pedestrian, heavy vehicle, etc.), road type and severity of injury (Creutzig et al 2012, Xia et al 2015, Lindsay et al 2011, Woodcock et al 2009). Transportation and land use are tightly connected such that the balance of benefits and co-harms in a shift to more active transport depends on infrastructure. A 50% increase in cycling in a city with an extensive cycling infrastructure would make only a small difference in injury rates compared to cities where bicycle lanes are less common. In cities in which cycling is uncommon, the safety-in-numbers effect of reduced injury incidence with increasing bicycling prevalence is likely to result principally from growing pressures to invest in safer bicycling infrastructure (Macmillan et al 2014, Jacobsen 2003).

### Diet

As globalization and related social, economic, and demographic shifts continue, populations generally undergo a nutrition transition marked by increased consumption of animal source foods, added sugar, and processed foods (Popkin and Hawkes 2016). Consistent associations are found between these diets and high levels of noncommunicable disease (NCD) and GHG emissions, and between diets containing mostly minimally processed plant foods, whole grains, and pulses and lower levels of NCD and GHG emissions (Jones et al 2016, Auestad and Fulgoni 2015, Hallström et al 2015, Tilman and Clark 2014). Among foods, red meat has the highest GHG emissions and has been associated with health conditions including cardiovascular disease (Pan et al 2012, Sinha et al 2009), stroke (Kaluza et al 2012), type 2 diabetes (Pan et al 2011, Micha et al 2010), and some cancers (Cho et al 2003, Cross et al 2007, Ma and Chapman 2009, Norat and Riboli 2001). Accordingly, dietary change presents an important opportunity for obtaining health-climate co-benefits.

However, relationships between overall diet healthfulness and reduced GHG emissions are somewhat inconsistent, in part because sugar and snacks are often found to have relatively low GHG emissions compared to animal sourced foods and even compared to fresh produce, especially when it is air freighted or grown in heated greenhouses (Jones et al 2016, Payne et al 2016, Auestad and Fulgoni 2015, Hallström et al 2015). The variation is also attributable to considerable heterogeneity in study designs and data sources.

We identified six model-based assessments of the health-climate co-benefits of diet scenarios. Two were global in scope and considered the relative health co-benefits of diet changes by region (Springmann et al 2016, 2017). One study focused on the US (Hallström et al 2017), and the three others provided estimates for the UK (Aston et al 2012, Friel et al 2009, Scarborough et al 2012), with the latter also providing a São Paulo, Brazil case study. Most focused on reducing meat consumption or livestock production in regions with high consumption patterns, although Springmann et al’s model (Springmann et al 2016, 2017) included regional analyses and developing countries.

All studies considered health co-benefits from reduction of exposures related to meat intake, which variously included saturated fat from animal sources, cholesterol, and red and processed meat products. Springmann et al (2016), (2017) and Hallström et al (2017) investigated diet more comprehensively by also considering exposures such as increasing consumption of plant foods and from substituting lower-emission alternatives for meat, total energy, refined sugar, and whole versus processed grain intake. Health outcomes included coronary heart disease in all cases and often diet-related cancers, stroke, and type 2 diabetes.

#### Approaches

All six diet studies explicitly included both climate and health benefits of changing diets, but with different approaches. Three of the studies began with a simultaneous consideration of both climate and health impacts of diets (i.e. behavior-focused). Aston et al assumed that the food system accounted for one third of UK GHG emissions, and that animal products are especially emissions intensive. They calculated the proportion of the UK population with different diets based on consumption of red and processed meat, then calculated the changes in GHG emissions and relative risk (RR) of NCDs if high consumers of animal foods had diets of low consumers. Springmann et al (2016) began with the assumption that animal based foods are both a major source of GHG emissions and NCDs, and that diet change could be ‘more effective than technological mitigation options for avoiding climate change.’ They then estimated the GHG emissions and RR of NCDs for four dietary scenarios that progressively excluded more animal-sourced foods. Hallström (2017) created counterfactual healthy alternative diets statistically associated with changes in the RR for three NCDs, calculating the GHG emissions of producing these diets and even of the health care system related GHG emission savings.

The other three studies began with climate change mitigation strategies (i.e. emissions focused). Friel et al 2009 modeled the effect of four strategies (technological changes and a 30% reduction in production in UK livestock industry) needed to reduce GHG emissions in the UK to meet official mitigation targets for 2030. They then estimated changes in population level intake of saturated fat and cholesterol, and the resulting effect on prevalence of ischemic heart disease and stroke. Scarborough et al (2012) used the UK CCC carbon budget diet scenarios, designed as a climate intervention, and then estimated population level health impacts. Springmann et al (2017) assumed that ‘GHG emissions related to food production will have to become a critical component of policies aimed at mitigating climate change,’ and modeled a climate change mitigation policy of consumption taxes on all food commodities based on their GHG emissions. They then calculated dietary and weight related RR of health outcomes.

#### Policy scenarios

Of the diet studies, only Springmann et al (2017) discussed specific policies for achieving the modeled diet changes. Acceptability of the modeled diets at the individual and population level was not dealt with in these studies. In general, the objective was to ‘explore a range of possible environmental [climate] and health outcomes...to encourage researchers and policymakers to act’ (Springmann et al 2016). Exploration of policies for achieving diet change and their acceptability of modeled diets is an area of active research and policy development (Garnett et al 2015), but no studies addressing these questions met our inclusion criteria.

#### Policy baselines

The policy scenarios in the included diet studies were often compared with actual dietary patterns as the baseline. For example, policy scenarios included sex-specific doubling of the proportion of vegetarians, a wider adoption of eating habits approximating the diets of those in the existing lowest quintile of meat consumption, or observed vegetarian and vegan intakes (Aston et al 2012, Springmann et al 2016). Another approach was to consider published dietary guidelines or food exposures for which there was strong evidence of a correlation with disease, or a combination of the above approaches (Hallström et al 2017). Baselines typically reflected existing diets or were based on UN Food and Agriculture Organization (FAO) forecasted diets.

#### Temporal scales

Diet modeling studies presented results for a single year (or in one case, two years) between 2010 and 2050.

#### Sources of GHG emissions estimates

The studies all used life cycle assessments (LCAs) from other sources, but selected and adjusted values to some extent to make them more appropriate. The health benefits per CO_2_e were highly dependent on the assumptions, methods, and data in the LCAs used as sources of CO_2_e per unit of the foods in the baseline and counterfactual diets. The temporal, spatial, and structural boundaries used in LCAs can have large effects on CO_2_e per unit of food, such as whether to include land use change or food waste. For example, only three of the studies (Springmann et al 2016, 2017, Halström et al 2017) incorporated wasted food; given that an estimated 30% of the global food supply is wasted, the impact on estimates can be considerable (FAO 2013).

#### Modeling considerations

There was considerable variation related to diet definitions, the relationship between diet and health outcomes, and the influence of diet on GHG emissions in the modeling choices and underlying assumptions of the reviewed studies. Policy scenario diets were defined *de novo* by using assumptions about health or climate benefits or both, or by modifying existing diets based on assumptions about effects on GHG emissions, or health, or both. Some studies defined them prior to the study according to climate change mitigation policies. There was also diversity in the extent of actual food intake exposures included in the models regarding components of meat or of meat products and inclusion of refined sugars and pulses. The most extensive model used was DIETRON; that is parameterized by total energy, fruit, vegetables, fiber, total fat, monounsaturated fatty acids, polyunsaturated acids, saturated fatty acids, trans fats, dietary cholesterol, and salt (Scarborough et al 2012).

Effects of diets, component foods, food compounds, or diet-related proximal risk factors (e.g. overweight and obesity) on health were evaluated in terms of the effect of change in the relative risk of non-communicable diseases (most frequently CHD, cancers, type 2 diabetes), or change in mortality, YLL, or DALYs. Climate impact was used to define diets alone or in combination with other parameters, and/or was modeled in terms of GHG emissions per diet, food unit, or production unit (e.g. livestock). Most studies did not include indirect feedbacks from diet change on climate in their models, for example the effect of land use change resulting from decreased consumption and production of animal foods (see below: co-harms). Hallström et al (2017) included the reduction in GHG emissions due to reductions in health care costs as a result of healthier diets.

#### Exposure–response function considerations

Population-attributable fractions (PAFs)/population impact fractions (PIFS) are used to estimate changes in morbidity and mortality due to scenario diets, based on diet or body weight risk factors from observational (correlational, cross-sectional) or experimental (randomized controlled trial) data from epidemiological studies, or meta-analyses of these data (Scarborough et al 2012, Springmann et al 2016, Springmann et al 2017, Hallström et al 2017).

#### Results

There was great variability in populations, definitions of diet components and diseases, risk estimates, and methods, making it difficult to directly compare health outcomes per CO_2_e avoided. Overall, however, the scenarios modeled in these studies yielded considerable reductions in chronic disease and mortality, and in GHG emissions. For example, Springmann et al (2016) estimated that a 25%–190% increase in fruit and vegetable consumption, and 56%–78% reduction in meat consumption could result in 5.1 million global deaths avoided from coronary heart disease, stroke, cancers, and type 2 diabetes, and a reduction of 11.4–8.1 Gt year^−1^ of food-related GHG emissions. They further estimated that shifting the global population to vegetarian diets, and increasing produce consumption by 54% would result in avoiding 8.1 million deaths and a reduction of 11.4–3.4 Gt year^−1^ of food-related GHG emissions by 2050. They estimated the economic benefits at $1–31 trillion, or 0.4%–15% of global GDP in 2050.

#### Relevance and inclusion of co-harms

It is important to note that while there are co-benefits for many foods, and actual and model diets, this is not universal. There can be tradeoffs, or co-harms, between climate and health effects. Whether diets generate co-benefits or co-harms depends on how they are defined, and their definitions in terms of climate and health vary greatly. For example, compared with the existing US diet, the 2010 diet recommended by the USDA for improved nutrition increased GHG emissions 12% (Heller and Keoleian 2015). The main reason was that reduced meat consumption was balanced by an increase in dairy consumption, and to a lesser extent by an increase in seafood, fruit, oils and vegetables. However, the health benefits of dairy can be obtained in foods without the potential health costs of dairy (not considered by the USDA), and the USDA-recommended vegan diet with excess caloric intake eliminated reduced emissions from the current diet by 53%, suggesting that reducing or eliminating dairy may be critical for avoiding co-harms from some recommended diets.

Existing diets that have co-benefits in terms of some component foods or compounds may have co-harms in terms of others, and this varies between diets. For example, Payne et al (2016) reviewed 16 studies, including 100 existing dietary patterns, almost all in the Global North. They evaluated the relationship between diets containing nutrients (which they independently estimated) that are bad for health (e.g. saturated fat, salt), and those that are good for health (e.g. micronutrients), and the GHG emissions of the diets. They found that the majority of diets with lower GHG emissions had higher sugar and lower micronutrients, and that these diets had equal or higher levels of mortality and non-communicable diseases.

Of the studies included in this review, only the Springmann et al 2016 analysis considered co-harms of diet change directly. That study considered the ways that the projected dietary change might result in under nutrition among vulnerable populations, concluding that the corresponding health benefits outweigh these harms. They recognized there were disparities in who would benefit and be harmed.

An important class of co-harm is rebound, an issue particularly pertinent to dietary considerations, and which was dealt with in very few of the studies reviewed. Rebound can result when savings in the food or healthcare systems due to diet change are invested in activities that generate GHG emissions or disease that take back some of the benefits of the modeled scenarios. Rebound can also be negative and function as a co-benefit by reinforcing the intended effect of the scenario by investing health care savings from improved diets in improving access to fruits and vegetables, or revegetation of rangeland no longer needed for animal production, to increase carbon sequestration. While rebound is, as noted, outside the scope of this review, it is an important consideration for future co-benefits studies.

## Discussion

Overall, despite the diversity in methods, scenarios, exposures, temporal scales, and other considerations, two important conclusions can be drawn from our review of health co-benefits studies. First, these studies consistently demonstrated that the health co-benefits of mitigation policies and technologies offset a significant portion of their implementation costs. Second, health co-benefits accrue sooner than the direct benefits of reducing GHG emissions. That is, in many instances, implementing some mitigation policies appears to make sense because of the improvements to population health even without considering the benefits for achieving climate policy.

Unfortunately, at this stage, meta-analyses of the literature are not possible because of the diversity of approaches and assumptions. The power of this research to support policy change would be increased with greater consistency (Jack and Kinney 2010, Remais et al 2014). This diversity reflects the range of questions being asked, the different scales at which analyses are being applied, and highlights the interest in estimates of the health co-benefits of possible climate mitigation policy choices.

However, some degree of diversity can be beneficial because local scale analyses that compare a limited set of policy choices are important for local decision makers to support choosing among specified mitigation options, and identifying those that maximize health co-benefits and greenhouse gas emission reductions. These studies must focus on the specific question(s) of interest. That the analyses might not have relevance elsewhere is a secondary and minor consideration.

The policy relevance of studies focusing on larger temporal and spatial scales, particularly those designed to explore the current or future health co-benefits associated with a change in air pollution, transportation, or diet, would be enhanced by agreeing on a limited set of population, health outcomes, scenarios, time slices, and discount rates. This is not to suggest limiting studies to a subset of the range of possible choices, but to recommend that studies at least model a consistent set of choices; doing so would promote meta-analyses and the possibility of adding results across several studies to estimate co-benefits over larger geographic scales.

### Modeling approaches determine modeling choices

The inherently interdisciplinary nature of health co-benefits analyses persists in limiting the availability of studies that meet the sort of rigor and credibility across physical and societal systems prescribed by authors who noted the apparent lack of policy traction (Jack and Kinney 2010, Nemet et al 2010, Remais et al 2014). While some have attempted systems-level credibility (Thompson et al 2014), the researcher’s perspective continues to dictate assumptions, data sources, sophistication, and comprehensiveness. In the short term, initiating analyses from a climate policy perspective (as in integrated assessment models) will likely continue to focus on the drivers of costs and GHG emissions, while initiating analyses from a health perspective will likely continue to examine a broader range of risks and outcomes.

### Construction of scenarios determines policy relevance

In the reviewed studies, scenarios were constructed from hypothetical ideals, concrete policies (existing or proposed), future socio-technological scenarios, expert opinion, global guidelines (e.g. WHO/FAO), or in collaboration with local stakeholders. Where specific policies (e.g. COP21) or recognized scenarios exist (e.g. RCPs), their use enhances relevance and comparability. Studies based on mitigation strategies dependent on individual level changes in health behaviors (e.g. some of the dietary and active transportation studies) are limited in their ability to inform current climate change mitigation policy because policy does not consider individual behavior change or health. However, proposed policies may be more effective in obtaining organizational support, and in achieving results, by including climate-health co-benefits. Potential health benefits may also incentivize individual behavior change and encourage shifts in institutional policies, such as those related to food procurement.

In developing new policies, stakeholder participation can be crucial. Participatory system dynamics modeling (Macmillan et al 2014) involves stakeholders in producing a supported ‘dynamic causal theory’ and addresses the interdisciplinary and interlinked nature of co-benefits research with systems-level representations. For example, causal loop diagrams (e.g. Macmillan et al 2014) and conceptual frameworks describe the model scope and causal theory assumed by the model and can assist with determination of policy levers for influencing desired health and emissions outcomes. Due to inherent complexity and uncertainty, modeling studies elucidate the complex interactions between policy, GHG emissions, and health rather than predict a particular outcome at a point in time (Macmillan et al 2014).

### Treatment of data gaps

Lack of data availability for some model inputs means that certain co-benefits or co-harms cannot be fully quantified. Modelers used a variety of techniques to address these data gaps. For instance, in air quality studies when comprehensive representative data were not available for particular countries or regions, concentration-response functions from epidemiological studies conducted in the US or other developed countries were used (e.g. West et al 2013). Also for example, Springmann et al (2016) collapsed categories from FAO data covering 110 regions and 32 food commodities and aggregated it to 107 regions and 16 commodities to match data availability for environmental and health analyses, and omitted global recommendations for food groups (i.e. fat, salt, whole grains, pulses) for which there were not adequate health data or recommendations. Hallström et al (2017) did not include diet-NCD links for which there was not the highest quality data pertaining to the RRs, so their result for GHG emissions reductions due to the health care effects of healthier diets was conservative. Sensitivity analyses were prevalent among studies in all three sectors to evaluate the influence of underlying sources of uncertainty and missing data (e.g. Sarofim et al 2017, Liu et al 2017, Xia et al 2015).

### Treatment of time lag

Modeling studies must make assumptions related to the temporal dynamics of emissions, exposures, and health outcomes. Policy implementation in reality can be gradual and incomplete. There is also a lag between policy implementation and resulting changes in exposure, and changes in exposure rarely result in immediate health impacts. For example, there is a temporal gap between changes in diet-related GHG emissions and associated health impacts. The former would occur within several years of population dietary change, as food production shifted to accommodate demand, while health effects could be delayed by decades.

Most studies did not attempt to address the temporal dynamics of policy, exposure, climate and health effects, or incorporated no lag time because evidence suggests the impact on quantified benefits is small (Thompson et al 2014). Taking diet as an example again, in most models the climate and health effects were assumed to be equivalent to the diets having been adopted for some time, or the effects of the diets happening all at once. Other models assumed the results would be obtained by a climate change mitigation target year. Some researchers addressed the lag by employing the concept of ‘committed impact’ (i.e. counting the long-term impact of a change in exposure). Creutzig et al (2012) modeled the ‘transitiondynamics’ of transportation policies to address implementation phasing, but still assumed instantaneous effects on health.

### Incorporating complexities in health exposures

Estimates of health co-benefits are sensitive to the source of relative risks applied, the age- and sex-specific granularity applied, and the range of exposure concentrations considered, especially when the exposure-response curve is uncertain at the high end of exposures. For example, for some health risks there may be no safe level exposure, but epidemiology data may be incomplete at the extremes, so models may either assume there is an exposure threshold below which there is no measurable health effect, or assume no threshold and test for sensitivity at the limits for which data are available (e.g. West et al 2013). Furthermore, baseline health status and likelihood of behavior modification can influence susceptibility to exposure and vary by population segment, which impacts the distribution of health co-benefits. Many studies did not address these complexities. However, Maizlish et al (2013) considered age- and gender-specificity of physical activity and health outcomes, as well as decreased population level variability in commute speed and active travel participation with increased prevalence of cycling and walking (Maizlish et al 2013). Relevance of these types of complexities is determined by the research and policy objectives.

### Interoperability between health models and integrative assessment models (IAMs)

Employing IAMs, such as those from the International Institute of Applied Systems Analysis, is a way for health co-benefits studies to expand on existing standardized models of large scale interactions among population, technology, socioeconomic factors, and emissions and link them to health outcomes. There are two main IAM approaches: IAMs that incorporate their own estimate of emissions to impacts, and IAMs that couple with a more comprehensive health impacts tool (pathway of emissions to concentrations to health). The former often uses simplified relationships of emissions to health impacts that neglect, for example, complex chemical nonlinearities (Thompson et al 2014). There is increasing sophistication in the tools used to link emissions directly to outcomes; however, there remains considerable disagreement between these approaches and caution is warranted in applying them beyond their context (Heo et al 2016). For both approaches, it would be helpful to increase the number and kind of health outcomes considered to more broadly reflect the range of health co-benefits that could arise. Increasing the interoperability between health and integrated assessment models would facilitate this inclusion.

### Guidelines for accurate and transparent health estimates reporting (GATHER)

The WHO GATHER (Stevens et al 2016) include definitions of technical terms (health indicator, health estimates, data inputs, and covariates) and a checklist pertaining to study population, data inputs and analyses, results, and discussion. GATHER maintains that items on this checklist should be specified alongside published health estimates to facilitate reasonable comparisons across time and between different populations, and appropriate use of health estimates in policy, planning, and monitoring. Specifically, ‘GATHER aims to define best practices for reporting of studies that synthesize information from multiple sources to quantitatively describe past and current population health and its determinants,’ similar to what health co-benefits modeling studies do, although co-benefits studies often project future impacts.

Models of health co-benefits of climate mitigation span a global range in populations and data sources and are often constrained by data availability. Utility, comparability and synthesis of these models therefore depends on interpretation of their results and limitations. GATHER provides a best practice and standard method of documenting population-level health-related indicators and determinants.

Overall, the studies reviewed, while they do not specifically mention GATHER, for the most part comply with the guidelines. One area in which co-benefits studies do not always adhere to the GATHER guidelines is in the treatment of uncertainty. GATHER requires a quantitative measure of uncertainty, including methods for calculating uncertainty and articulation of which sources of uncertainty are and are not accounted for. Sensitivity analyses are prevalent but not ubiquitous among health co-benefits of climate mitigation studies.

### Valuation of health co-benefits

The role of valuation of health co-benefits in the policy discourse and methods for estimating monetized health benefits have been described and discussed (Bell et al 2008, Springmann et al 2016a, Nemet et al 2010). Valuation approaches include: value of statistical life (used in cost/benefit analyses), value of life years lost with mortality analysis by age segmentation, benefits transfer approach, cost of illness (quantifies direct costs of morbidity), and willingness to pay (to reduce mortality risk), among others. Valuation approaches range from very narrow estimating only avoided health costs to very broad including net societal benefits. When accounting for health impacts in evaluating cost effectiveness of mitigation options, both the scope of health impacts modeled and the valuation approach must be considered.

### Equity considerations

Equity is a major pillar of the causes, impacts, and solutions to climate change, yet few studies have considered the social distribution of health co-benefits. The study by Springmann et al (2016) on diet co-benefits is an exception. While some air quality models consider regional equity, there is a dearth of studies addressing socioeconomic dimensions, and we found no transportation studies that modelled the effects on health of climate mitigation through an equity lens.

The direct effects of climate mitigations are not experienced evenly, and the co-benefits and co-harms of climate mitigation policies may not be distributed equitably. Specifically, there is a pattern of inequity between the production of total and food system GHG emissions, and the vulnerability to climate change. Diet changes that would reduce GHG emissions could facilitate a shift in resources from wealthy populations to less wealthy populations, with reduced consumption in the former allowing for some increased consumption in the later, improving health in both. This was explicitly addressed only by Springmann et al (2016), whose scenarios reduced or eliminated (vegan diet) the food-related GHG emissions gap between high per capita emissions in the Global North and low per capita emissions in the Global South. Policy interventions to shift costs of diets in relationship to climate and health benefits, as explored in Springmann et al (2017), result in regressive outcomes. Those authors suggest complementary policy strategies to improve equity, such as excluding health-promoting foods from taxation and providing compensation to those most affected.

## Conclusions

As noted throughout this review, while the studies of the benefits of air quality, transportation, and diet mitigation policies consistently report health co-benefits, meta-analyses and syntheses of results are stymied by the diversity of approaches, modeling methods, policy scenarios, assumptions, time slices, and evaluation metrics. Increasing consistency across the air quality, transportation, and diet studies would begin to provide more comprehensive estimates of health co-benefits and to explore potential synergies and dis-benefits of baskets of mitigation options.

To a large extent, the reviewed literature achieved many of the recommendations of Jack and Kinney (2010) in that the studies reported scenarios and relationships of emissions to health-determinants, and quantified health outcomes. However, reporting is clearly insufficient if the literature is to fulfill its potential for having a significant policy impact, particularly at larger geographic and political scales; greater consistency is needed to conduct syntheses and meta-analyses of the health co-benefits of mitigation policies. Achieving this consistency is critical as nations are developing baskets of mitigation options to achieve their NDCs to the Paris Agreement. As choices are made, policymakers will be ill informed without a holistic view of the costs and benefits of the options. Incorporating a larger basket of health outcomes would provide more accurate estimates of the magnitude of possible benefits. Further, as noted by Remais et al (2014), considering dis-benefits also would increase understanding of positive and negative aspects of mitigation policies.

This is not to say that scientific knowledge is the only or even the primary driver of policymaking (Verboom et al 2016). The processes by which policy and decision-makers assess and use information is complex. Polices need to take into consideration not only scientific evidence, but also competing priorities, interests, and values, and perceptions of equity, fairness, and ethics (Bowen and Zwi 2005), among other considerations. Iterative engagement between researchers and policymakers increases the capacity of policy makers to assess, evaluate, and use data in support of complex-policy interventions, and the capacity of researchers to provide policy-relevant results (Langlois et al 2016). Increased availability and use of simplified, universal models that facilitate rapid calculation, such as the greenhouse gas policy assessment model (GHG-PAM) developed by Liu et al (2017), could help align scientific insight to policy-making needs and realities.

The literature can be roughly divided into studies that focus on quantifying the health co-benefits of a specific, local mitigation policy, which generally are concerned with short-term benefits; and those that focus on larger geographic and temporal scales. Local scale studies will increasingly be needed to inform policy-makers of the benefits associated with specific policy recommendations, to provide balanced estimates of the net cost of these policies and to help policymakers choose among sets of mitigation options. Because the scenarios used are in response to policy-maker needs, diversity will and must continue. However, agreeing on comparable health outcomes, the concentration-response relationships, and approaches to estimating the economic benefits would increase the policy relevance of health co-benefits research.

There is also a significant opportunity for national and regional studies to use comparable choices to enable synthesis and more robust quantifications of health co-benefits. Again, increased consistency in the health co-benefits considered, the concentration-response relationships employed, and approaches to estimating the economic benefits would improve comparability and bring together emissions-focused and behavior-focused approaches (figure 2). In addition, modelers can develop a limited set of scenarios and time slices to explore as part of their projections of health co-benefits; additional scenarios may be of interest to address the study questions. We recommend that a few scenarios be included in all studies with a view toward synthesis and meta-analysis. Specifically, projections done through 2020 should focus at least on 2030 and projections done through 2040 should focus at least on 2050, and all projections should include a normative scenario that combines marker scenarios RCP 2.6 and the sustainability pathway in the Shared Socio-economic Pathways (SSP1) (O’Neill et al 2017), as this will be the closest to achieving the Paris Agreement (Riahi et al 2017), or a scenario consistent with the underlying drivers (e.g. SRES B1 and SRES A2, respectively). A high emissions scenario such as RCP 6.0 or 8.5 and Regional Rivalry in the SSPs (SSP3) would be a scenario with high population and high emission growth that could be used as a counterfactual for projections past 2050. Using similar temporal and spatial scales as employed in integrated assessment models means the costs of mitigation policies from these models could be compared with the health co-benefits (e.g. West et al 2012). Achieving this could be promoted by partnering with the integrated assessment modelers. Doing so would increase the complexity of health co-benefits analyses and their potential usefulness.

Another increasing area of interest is to understand the equity dimensions of mitigation policies and to whom the health co-benefits accrue. It is easy to imagine that some air quality mitigation policies could provide greater benefits to communities downwind of coalfired power plants, and that increasing active transport could benefit homes along major transportation routes if vehicular traffic is reduced. Reducing vehicle emission standards would further benefit these communities. Expanding the health co-benefits literature to consider particularly vulnerable communities and populations would help in form estimates of the extent to which mitigation policies also would have positive (or negative) equity dimensions.

These recommendations are within the context that model diversity itself has benefits (Ebi and Rocklov 2014). There is broad diversity across integrated assessment models used to estimate the costs of mitigation, but also sufficient consistency that model results can be compared and summed in some instances.

As the literature demonstrates, mitigation policies are very likely a ‘win-win,’ improving health in the shorter term while decreasing the magnitude of climate change-related health risks later in the century. Taking the health co-benefits into account provides more comprehensive estimates of mitigation policy costs and benefits, and may increase the political feasibility of mitigation policies because these health benefits are often significant, local, and immediate, accruing well before the climate benefits of reduced GHG emissions. Leveraging estimates of nearer-term, more proximal health benefits of climate mitigation policies and technologies is an opportunity to support policy uptake and implementation.

## Supplementary Material

Supplementary data
