# Supplementary material for: Ancillary health effects of climate mitigation scenarios as drivers of policy uptake: a review of air quality, transportation and diet co-benefits modeling studies
Source: Environ Res Lett. Author manuscript; Available in PMC 2024 Apr 11. (PMC11007749; doi:10.1088/1748-9326/aa8f7b)
Supplement: Supplementary data [file NIHMS1953501-supplement-Supplementary_data.pdf]

Supplementary Table 1 - Search terms and synonyms

Climate mitigation	climate mitigation, emissions, greenhouse gases
Health co-benefits	health co-benefits, ancillary benefits, health impact, premature mortality, deaths
Transportation	transportation, transport, walking, cycling, motor vehicle, urban transport, vehicle transport, physical activity
Air quality	air quality, air pollution, pollution, clean power, PM2.5
Diet	diet, meat consumption, food, meat

Supplementary table 2 – Health co-benefits of mitigation modeling studies - air quality

	Scenarios and Policy Relevance	Associated Change in Health Related Exposure	Health Outcomes Estimated	Baseline & Time Period	Study Location(s)	Change in Risk	Change in DALYs	Change in YLLs	Change in Mortality (deaths unless otherwise specified)	per CO2 Equivalent Averted	Monetized Benefits
**Markandya et al. 2009**	50% global CO2 reductions over 1990 levels by 2050 consistent with The First Report of the Committee on Climate Change (UK, 2008) and Working Group III of IPCC	(1) 4-10 μg/m^3 reduction in $PM_{2.5}$<br>(2) 0.3-10 μg/m^3 reduction in $PM_{2.5}$	Deaths due to:<br>(A) Cardiorespiratory disease<br>(B) Lung cancer<br>(C) Acute respiratory infections	BAU; 2030	EU, India, China	Not presented	Not presented	(1) A: -338 to -881, B: -39 to -72, C: -21 to -572 per million<br>(2) A: -78 to -881, B: -26 to -91, C: 0 to 572 per million	(1) -32 to -62 per million<br>(2) -10 to -62 per million	(1) 6522 million tonnes in 2030<br>(2) 6751 million tonnes in 2030	(1) 5.99 to 48.98 US$ per tonne CO_2 averted<br>(2) 1.82 to 45.94 US$ per tonne CO2 averted
**Gilmore et al. 2010**	500 MW sodium-sulfur battery charged during off-peak times of day and discharged during peak times replacing 4 hours of electricity generation from a (1) distillate fuel peaking plant or (2) natural gas peaking plant	(1) 0.12 μg/m^3 reduction in daily mean $PM_{2.5}$, increase of 1.0 ppb in daily mean O_3<br>(2) 0.03 μg/m^3 reduction in daily mean $PM_{2.5}$, 0.85 ppb increase in daily mean O_3	Premature mortality	per year	New York City	Not presented	Not presented	Not presented	(1) -9 per year, +7 per year<br>(2) -3 per year, +6 per year	Presented as CO_2 emission factors in g/kWh for a number of charging plant types	(1) $0.045/kWh, net cost<br>(2) $0.17/kWh, net cost
**Anenberg et al. 2012**	14 methane and black carbon mitigation measures consistent with United Nations Environment Programme and World Meteorological Organization 2011 integrated assessment of increased implementation of existing methane and black carbon mitigation	(1) 0.01-0.12 μg/m^3 reduction in $PM_{2.5}$, 2.82-4.09 ppb reduction in O_3<br>(2) 0.15-3.59 μg/m^3 reduction in $PM_{2.5}$, 4.00-9.95 ppb reduction in O_3<br>(3) 0.22-4.92 μg/m^3 reduction in $PM_{2.5}$, 3.92-11.0 ppb reduction in O_3	(A) $PM_{2.5}$ cardiopulmonary and lung cancer deaths<br>(B) O_3 respiratory deaths	IEA projections and already agreed upon mitigation policies 2005; 2030	Global	Not presented	Not presented	(1) A: 0.12 to 0.59, B: -0.61 to -0.94 million per 2030 projected population<br>(2) A: -11.8 to -14.9, B: -1.15 to -2.54 million per 2030 projected population<br>(3) A: -16.2 -20.5, B: -1.06 to -2.81 million per 2030 projected population	(1) A: -0.02 to -0.06, B: -0.07 to -0.10 million per 2030 projected population<br>(2) A: -1.39 to -1.74, B: -0.13 to -0.28 million per 2030 projected population<br>(3) A: -1.93 to -2.42, B: -0.12 to -0.31 million per 2030 projected population	Not presented	Not presented
**Crawford-Brown et al. 2012**	77% reduction in CO_2 emissions throughout economy via policies being considered as part of Nationally Appropriate Mitigation Actions	(1) 10 μg/m^3 reduction in PM_{10} (38%)<br>(2) 7.2 ppb reduction in O_3 (12%)	Mortality, non-fatal disease cases (hospital admissions and long term health care)	Baseline growth to 2050; 2010-2050	Mexico	Approximately 0.004 reduced lifetime excess probability of premature death	Not presented	Not presented	(1) -2252/year average annual total between 2010 and 2050<br>(2) -466/year average annual total between 2010 and 2050	1.2 $GtCO_2$ cumulative reduction	(1) $351M 2020 USD/year<br>(2) $246M 202 USD/year
**Patridge and Gamkhar 2012**	Replacement of electricity from a coal-fired plant with electricity from a plant using renewable energy	PM_{10} intake fractions specified in Zhou et al. 2006	Premature mortality, chronic bronchitis cases, hospitalizations	Marginal coal fired station compliant with existing regulations; per year	China	Not presented	Not Presented	Not presented	0.6 to 2.3 cases avoided per year per marginal plant	Not presented	(A) 1.6M RMB 2007<br>(B) 88,000 RMB 2007<br>(C) 11,200 RMB 2007
**Shindell et al. 2012**	14 measures targeting methane and BC emissions that reduce projected global mean warming ~0.5°C by 2050.		Premature mortality	RCP8.5; 2030, 2050, 2100	Global				0.7 to 4.7 million annual premature deaths	-0.77 mW/m^2 in 2030	CH_4 measures: US148 billion; BC Tech measures: $3717; BC reg measures: $1425
**West et al. 2012**	Total CH_4 reductions of 75, 125, and 180 Mton/year in 2030 described relative to IIASA Maximum Feasible Reduction, and IEA and US EPA benchmarks	(1) 0.7 ppb reduction in global average O_3<br>(2) 1.3 ppb reduction in global average O_3<br>(3) 2.0 ppb reduction in global average O_3	(A) Total mortality<br>(B) Cardiovascular and respiratory mortality	IIASA Current Legislation (CLE) scenario; 2005-2030	Global	Not presented	Not presented	Not presented	(1) Not presented<br>(2) A: -2.01 to -6.89 per million per year in 2030, B: -1.14 to -4.41 per million per year in 2030<br>(3) Not presented	(1) 75 Mton CH_4 /year<br>(2) 125 Mton CH_4 /year<br>(3) 180 Mton CH_4 /year	$50-$380 USD per tonne CO_2
**Chen et al. 2013**	(1) 1.35% overall total energy conservation by major industries in Taiwan and (2) 5,630,944 kWh savings at Taipei City Hall in 2009 as a result of Taiwan Bureau of Energy guidelines requiring at least 2% improved energy efficiency per year	Not presented; a simple rollback model was used to estimate changes in exposure concentrations as a component of the Air Resource Co-Benefits (ARCoB) model employed	All-cause mortality, all-cause outpatient cases, all-cause emergency cases, respiratory hospital admissions, heart disease hospital admissions	Without actual energy efficiency improvements; 2009	Taiwan	Not presented	Not presented	(1) -3,478 total in 2009<br>(2) -2.62 total in 2009	(1) -311 total in 2009<br>(2) -0.234 total in 2009	Not presented	(1) $10.34M USD averted medical expenditure<br>(2) $9,031 USD averted medical expenditure
**Crawford-Brown et al. 2013**	Generalized scenarios of fractional GHG emission reductions from uniform reduction measures across all sectors where (1) all nations or (2) only Annex I nations reduce emissions	50% reduction in PM_{10} ambient air concentrations (equal to 50% of PM_{10} emission reductions & 50% percent reduction in GHG emissions) by region	Morbidity, cardiovascular and respiratory mortality	Unspecified	Global	(1) 0.0004 reduction in excess lifetime mortality risk for 80% GHG reduction<br>(2) 0.0024 reduction in excess lifetime mortality risk for 80% GHG reduction	Not presented	Not presented	(1) -120,000 per year for 80% GHG reduction<br>(2) -72,000 per year for 80% GHG reduction	Fractional GHG reduction	$2.5 USD per tonne GHG emissions reduced
**Rafaj et al. 2013**	60% global GHG emissions reductions over 1990 levels in 2050	36%-63% reductions in average ambient $PM_{2.5}$ concentration	(A) Statistical life expectancy (due to $PM_{2.5}$)<br>(B) Premature mortality (due to O_3)	No further climate or air pollution policies implemented after 2010; 2005-2050	Global and EU, China, India and US	Not presented	Not presented	Not Presented	(A) Increased life expectancy of 1.2-30 months in 2050<br>(B) -80,000 per year	Approximately 32 $GtCO_2$/yr in 2050	Not presented
**Rao et al. 2013**	(1) No additional legislation beyond 2005, and (2-5) scenarios of increasing stringency involving sets of air pollution, climate, and energy policies presented in Global Energy Assessment and referencing Conference of Parties in Copenhagen, UN call for universal access to modern energy, WHO recommended AQG value, and technological feasibility	Global PM2.5 of:<br>(1) 50.3 μg/m^3<br>(2) 34.0 μg/m^3<br>(3) 26.0 μg/m^3<br>(4) 15.7 μg/m^3<br>(5) 12.3 μg/m^3	Cardiopulmonary disease, lung cancer, acute lower respiratory infection, chronic obstructive pulmonary disease, and ischemic heart disease due to outdoor and household air pollution	2005 global baseline energy and GHG emissions described by Riahi et al. 2012, which is similar to RCP 8.5; 2030	Global	Not presented	(1) 50% increase over 2005 in 2030 from outdoor air pollution, (2) 30% increase over 2005 in 2030 from outdoor air pollution, (3) approx. equal to 2005 in 2030, (4) approx.. -5M from outdoor air pollution, (5) -11M from outdoor air pollution	Not presented	Not presented	Not presented	Not presented; policy costs reported without taking into account health-related savings
**West et al. 2013**	Representative Carbon Pathway 4.5 (RCP4.5): global carbon price across all sectors resulting in CO_2 concentration of 525 ppm in 2100	(1) 2.4 μg/m^3 reduction in $PM_{2.5}$<br>(2) 8.1 ppb reduction in O_3 in 2100	Premature mortality from cardiopulmonary disease and lung cancer (due to $PM_{2.5}$) and chronic respiratory mortality (due to O_3)	Reference scenario for RCP4.5 (REF); 2000-2100	Global, by region	Not presented	Not presented	Not presented	(1) 2030: -0.4M/year, 2050: -1.1M/year (2050), 2100: -1.5M/year<br>(2) 2030: -0.9M/year, 2050: -0.2M/year, 2100: -0.7M/year	Not presented, but annual total CO_2 reduction is used in calculation of marginal co-benefits	$50-$380 USD per tonne of CO_2
**Zapata et al. 2013**	Measures included in California's Global Warming Solutions Act of 2006, California Assembly Bill 32 (AB 32), categorized by sector into five levels of cumulative implementation: (1) Industrial, (2) + Electric and natural gas, (3) + Agricultural, (4) + On-road mobile sources, (5) + Other mobile	3-10% reduction in 24-h average $PM_{2.5}$ exposure	Mortality	BAU (without AB 32), 2030	California, statewide and by basin	Not presented	Not presented	Not presented	(5) -880 per year	Not presented	$3.9B-$5.4B from mortality change

	Scenarios and Policy Relevance	Associated Change in Health Related Exposure	Health Outcomes Estimated	Baseline & Time Period	Study Location(s)	Change in Risk	Change in DALYs	Change in YLLs	Change in Mortality (deaths unless otherwise specified)	per CO2 Equivalent Averted	Monetized Benefits
Balbus et al. 2014	10 options across three sectors (transportation, buildings, power plants) with corresponding changes in activity levels that each would account for one "US wedge" amounting to 19 $GtCO_2$ cumulative reduction over 50 years; (1) combine 2 transportation wedges, (2) combine 2 buildings wedges, (3) combine 3 power plant wedges, (4) combine 2 buildings and 5 power plant wedges	$PM_{2.5}$ intake fraction	(A) Premature mortality, all-cause >29 years, (B) premature mortality, all-cause <1 year, asthma-related ER visits	Per wedge; 2020	US	Not presented	Not presented	Not presented	(1A) -1,520 to -931 /year; (1B) -689 to -432 /year<br>(2A) -689 to -432 /year; (2B) -2,484 to -1,367 /year<br>(3A) -2,970 to -1,884 /year; (3B) -1,362 to -889 /year<br>(4A) -9,071 to -5,166 cases/year; (4B) -4,277 to -2,422	Results presented per "US wedge" (19 GtCO2 cumulative reduction over 50 years)<br>(1) 2 wedges<br>(2) 2 wedges<br>(3) 3 wedges<br>(4) 7 wedges	$5,900M to $56,000M per wedge
Thompson et al. 2014	(1) Clean Energy Standard (CES), (2) transportation policy targeting passenger and heavy duty vehicles, (3) Cap-and-Trade	(1) 0.58 ppb population-weighted reduction in O_3, 0.97 $\mu g/m^3$ reduction in population-weighted $PM_{2.5}$<br>(2) 0.99 ppb population-weighted reduction in O_3, 1.16 $\mu g/m^3$ reduction in population-weighted $PM_{2.5}$<br>(3) 0.21 ppb population-weighted reduction in O_3, 0.56 $\mu g/m^3$ reduction in population-weighted $PM_{2.5}$	Increased mortality risk due to O_3 and $PM_{2.5}$	BAU with no carbon emissions constraints; 2030	US	Not presented	Not presented	Not presented	Presented in supplementary materials for each of 6 (O_3) and 2 ($PM_{2.5}$) published concentration response functions	500M tonne reduction over 2006 emissions in 2030	(1) $134B-$334B; $254/tCO2<br>(2) $210B-$340B<br>(3) $14B-$124B; $140/tCO2<br>overall, 26–1,050% of costs offset
Driscoll et al. 2015	Two Bipartisan Policy Center scenarios: (1) low stringency, power plant improvements, (3) high stringency, cost of carbon, and one Natural Resources Defense Council scenario: (2) moderate stringency, electricity sector improvements for comparison to the Clean Power Plan goals	Annual average $PM_{2.5}$ $\mu g/m^3$ and annual average peak summertime O_3 ppb	Total premature deaths, total respiratory and cardiovascular hospitalizations, total nonfatal heart attacks avoided	2013 Annual Energy Outlook forecast including fully implemented current EPA clean air policies; 2020	US	Not presented	Not presented	Not presented	(1) 10 per year<br>(2)-3,500 per year<br>(3) -3,200 per year	(1) 2.2% reduction compared to 2020 reference (approx. 50 Mt/yr)<br>(2)23.6% reduction compared to 2020 reference (approx. 500 Mt/yr)<br>(3) 39.8% reduction compared to 2020 reference (approx. 825 Mt/yr)	Not presented
Garcia-Menendez et al. 2015	(1) Global carbon tax to achieve total radiative forcing of 4.5 W/m^2 in 2100 (POL4.5), (2) global carbon tax to achieve total radiative forcing of 3.7 W/m^2 in 2100 (POL3.7)	(1) 2050: -0.5 ppbv 8-hour O_3 max, -0.2 $\mu g/m^3$ $PM_{2.5}$; 2100: -2.9 ppbv 8-hour O_3 max, -1.0 $\mu g/m^3$ $PM_{2.5}$<br>(2) 2050: -0.5 ppbv 8-hour O_3 max, -0.3 $\mu g/m^3$ $PM_{2.5}$; 2100: -2.6 ppbv 8-hour O_3 max, -1.2 $\mu g/m^3$ $PM_{2.5}$	Avoided deaths, life years saved	No mitigation, continued economic growth and unconstrained emissions to 10 W/m^2 in 2100; 2050 & 2100	US	Not presented	Not presented	(1) 2050: 570,000; 2100: 1,300,000<br>(2) 2050: 620,000; 2100: 1,400,000	(1) 2050: -11,000 deaths; 2100: -52,000 deaths<br>(2) 2050: -13,000 deaths; 2100: -57,000 deaths	(1) 330 ppm<br>(2) 370 ppm	Offset of 26-1,050% of cost of US carbon policies
Saari et al. 2015	(1) Clean Energy Standard, (2) Cap-and-Trade	(1) -2.9% PM_{10}, -4.9% $PM_{2.5}$<br>(2) -2.5% PM_{10}, -4.6% $PM_{2.5}$	Avoided deaths presented; additional endpoints included in BenMAP	BAU (emissions grow to 6200 mmt); 2030	US, by region	Not presented	Not presented	Not presented	(1) -36,591 in 2030<br>(2) -24,917 in 2030	500 mmt	(1) $8/$tCO_2$; 5% offset<br>(2) $6/$tCO_2$; 110% offset
Schucht et al. 2015	Climate mitigation limiting global temperature to 2°C by 2100 with full implementation of current legislation by 2030 (RCP2.6)	Annual average population weighted $PM_{2.5}$ approximately 500 $\mu g/m^3$ * million population and O_3 approximately 3,000,000 ppb days * million population	Premature deaths, Respiratory hospital admissions, Minor restricted activity, Respiratory medication use, Life years lost, Infant mortality, Chronic bronchitis, Cardiac hospital admissions, Restricted activity days, Respiratory medication use, Lower respiratory symptoms	No climate policy consistent with RCP8.5; 2050	Western and Central & Eastern Europe	Not presented	Not presented	-700,000/year	-41,100/year	Presented per emission and radiative forcing only	62,000M to 127,000M €/year; 85% offset to additional cost of climate policy
Sabel et al. 2016	Adopted urban climate mitigation policies related to transportation, buildings, and energy	Air pollution listed in "Additional file 1", Table A2a, physical activity listed in "Additional file 1", Table A2b	Mortality, morbidities, DALYs presented in "Additional file 1", Table A3	BAU; 2010, 2020	Kuopio, Finland; Rotterdam, Netherlands; Stuttgart, Germany; Basel, Switzerland; Thessaloniki, Greece; Xi'an, China; Suzhou, China	Various (presented in "Additional file 1", Table A3)	Various (presented in "Additional file 1", Table A3)	Various (presented in "Additional file 1", Table A3)	Various (presented in "Additional file 1", Table A3)	Various (presented in "Additional file 1", Table A3)	Not presented; instead measures of "economic wellbeing" presented in "Additional file 1" Table A4c
Shindell et al. 2016	Energy emissions reductions avoiding 0.05°C to 0.07°C warming in 2030 and 0.25°C in 2100 assuming a constant rate of decrease between 2015 and 2050 (63% reduced energy sector emissions)	$PM_{2.5}$, O_3	Premature mortality	RCP8.5; 2030, 2050, 2100	US	Not presented	Not presented	Not presented	-22,000 /year; -175,000 cumulative deaths 2015-2030	Not presented	Not presented
Thompson et al. 2016	(1) Clean Energy Standard (CES)(2) Cap-and-Trade. Each is implemented either nationally or applied to 17 states located in the Northeastern US.	$PM_{2.5}$, O_3	Increased morbidity and mortality risk due to O_3 and $PM_{2.5}$ Morbidities include: respiratory and cardiovascular hospitalizations, respiratory symptoms, total nonfatal heart attacks avoided	BAU with no carbon emissions constraints; 2030	US	Not presented	Not presented	Not presented	Presented for 2 (O_3) and 2 ($PM_{2.5}$) published concentration response functions. (1) 25 deaths avoided per 1 M tCO_2 avoided (2) 14 deaths avoided per 1 M tCO_2 avoided	500M tonne reduction over 2006 emissions in 2030	(1) 180% cost offset; $148/tCO2;<br>(2) 826% cost offset; $80/tCO2
Sarofim et al. 2017	Mitigation of marginal ton of CH_4 emissions	(1) -13 ppt 24-h average surface O_3<br>(2) -3.0 to -3.6 ppb 1-h maximum O_3	Premature short-term respiratory mortality	2010; 70 year period	Global	Not presented	Not presented	Not presented	(1) -591 deaths<br>(2) -239 deaths	ton CH_4 reduction	(1) US $1,775<br>(2) US $790

	Scenarios and Policy Relevance	Associated Change in Health Related Exposure	Health Outcomes Estimated	Baseline & Time Period	Study Location(s)	Change in Risk	Change in DALYs	Change in YLLs	Change in Mortality (deaths unless otherwise specified)	per CO2 Equivalent Averted	Monetized Benefits
**Liu et al. 2017**	(1) Industrial structure dominated, (2) technology dominated, (3) Integrate carbon reduction defined according to parameters of Suzhou low carbon development planning	(3) 44% decrease in total fine PM annual intake (-1123 kg)<br>(2) 41% decrease in total fine PM annual intake (-1052 kg)<br>(1) 5% decrease in total fine PM annual intake (-132 kg)	Morbidity and mortality from cardiovascular and pulmonary disease	BAU; 2020	Suzhou City, China	Not presented	(1) -6.0 kDALY<br>(2) -48.2 kDALY<br>(3) -116.0 kDALY (-44.1%)	Not presented	Not presented	(1) -13 million tons<br>(2) -117 million tons<br>(3) -124 million tons	Not presented

Supplementary table 3 – Health co-benefits of mitigation modeling studies - transportation

	Scenarios and Policy Relevance	Associated Change in Health Related Exposure	Health Outcomes Estimated	Baseline & Time Period	Study Location(s)	Change in Risk	Change in DALYs	Change in YLLs	Change in Mortality (deaths unless otherwise specified)	per CO2 Equivalent Averted	Monetized Benefits
**Woodcock et al. 2009**	Scenarios based on Visioning and Backcasting for Transport (VIBAT) study conducted to inform the Greater London Authority 2025 target of 60% reduction in cross-sector emissions:<br>(1) Lower-carbon-emission motor vehicles<br>(2) Increased active transport<br>(3) Towards sustainable transport	km walked and biked per person per year:<br>(1) No change in walking (km per person per year), No change in biking (km per person per year), -0.4 to -11.4 μg/m^3 $PM_{2.5}$<br>(2) 33%-146% increase in walking, 240%-804% increase in biking, -0.5 to -14.9 μg/m^3 $PM_{2.5}$<br>(3) 33%-146% increase in walking, 240%-804% increase in biking, -0.8 to -18.1 μg/m^3 $PM_{2.5}$	Cardiovascular disease, cerebrovascular disorder, diabetes, cancer rates, dementia-Alzheimer's, depression, acute respiratory infections, road traffic injuries, mortality, obesity, overweight	BAU, 2030	London, Delhi	Not presented	(1) -160 to -1696 per million population per year<br>(2) -7,332 to -12,516 per million population per year<br>(3)-7,439 to -12,995 per million population per year	(1) -1,696 to -5,188 per million population per year<br>(2) -5,188 to -10,969 per million population per year<br>(3) -5,295 to -11,448 per million population per year	(1) -17 to -74 per million population per year<br>(2) -511 to -530 per million population per year<br>(3) -532 to -541 per million population per year	(1) 0.66 to 0.73 tonnes<br>(2) 0.40 to 0.69 tonnes<br>(3) 0.36 to 0.45 tonnes	Not presented
**Lindsay et al. 2011**	Replacing light vehicle transport with bicycle transport for (1) 1%, (2) 5%, (3) 10%, and (4) 30% of short (<7km) trips	Air pollution, motor vehicle-cyclist collisions, energy expenditure over baseline resting	(A) Air pollution deaths, restricted-activity days, acute cardiac and respiratory admissions, collision hospitalizations, (B) cyclist fatalities, (C) mortality reductions due to physical activity, body fat	Annual	New Zealand	Not presented	Not presented	Not presented	(1) A: -1.1, B: 3.5, C: -20.5 annually<br>(2) A: -5.6, B: 5.0, C: -116.5 annually<br>(3) A: -11.3, B: 6.1, C: -165.3 annually<br>(4) A: -33.9, B: 9.1, C: -716.2 annually<br>17.5% mortality reduction per 1,000 commuter cyclists per year	(1) 10,735 tonnes<br>(2) 53,676 tonnes<br>(3) 107,351 tonnes<br>(4) 322,054 tonnes	$1,050,000 per 1,000 commuter cyclists (20-64 yrs.) per year
**Rojas-Rueda et al. 2012**	Within Barcelona City: (1) 20% car trip trips reduction replaced 100% by bike, (2) 40% car trips reduction replaced 100% by bike, (3) 20% car trips reduction replaced 50% by bike and 50% by public transportation, (4) 40% car trips reduction replaced 50% by bike and 50% by public transportation<br>Between inner-city and Barcelona metropolitan region: (5) 20% car trip trips reduction replaced 100% by public transportation, (6) 40% car trips reduction replaced 100% by public transportation, (7) 20% car trips reduction replaced 20% by bike and 80% by public transportation, (4) 40% car trips reduction replaced 20% by bike and 80% by public transportation	Physical activity (walking and biking), traveler exposure to $PM_{2.5}$, -0.07 to -0.26 μ/m^3 $PM_{2.5}$ (general population exposure), road traffic fatality	Mortality	BAU, 2009	Barcelona City, Spain	Not presented	Not presented	Not presented	Total net change in deaths per year, months of life expectancy gained:<br>(1) -33.06, 6.5<br>(2) -66.12, 6.5<br>(3) -21.88, 4.7<br>(4) -43.76, 4.7<br>(5) -20.07, 2.8<br>(6) -40.15, 2.8<br>(7) -49.25, 4.7<br>(8) -98.5, 4.7	203,251 t/CO2 per year	Not presented
**Creutzig et al. 2012**	Four increasingly ambitious climate city-specific policy packages defined iteratively with stakeholder engagement	Transport related physical activity	Life savings	BAU 2010, 2040	(A) Barcelona<br>(B) Freiburg<br>(C) Malmö<br>(D) Sofia	Not presented	Not presented	Not presented	Statistical lives saved/year due to walking, statistical lives saved/year due to biking:<br>(A) 194-708, 16-162<br>(B) 2.5-11.8, 15-234<br>(C) 27-74, 51-140<br>(D) -93 to -15, 20-117	Change in tCO_2 per year per capita:<br>(A) -0.8 to -1.2<br>(B) -0.9 to -1.3<br>(C) -0.6 to -1.3<br>(D) -0.8 to -1.5	Not presented
**Grabow et al. 2012**	Substitution of all short car trips of 5 miles or less round trip from April to October with alternative means of transportation such as walking, biking, or mass transit. The model for health benefits from transport related physical activity assumed that 50% of these short trips were taken by bicycle	Changes in exposure to $PM_{2.5}$ and O_3; changes in exposure to transport-related physical activity	Air Pollution: Overall mortality, asthma exacerbations, chronic bronchitis, hospital admissions, acute myocardial infarctions, acute and chronic respiratory infections, upper and lower respiratory infections, work -loss days, and school-loss days; Transport Related Physical Activity: All-Cause Mortality	2002	11 largest cities in Upper Midwest United States	Not presented	Not Presented	Not presented	Avoided 1,295 deaths (608 avoided due to improved air quality and 687 deaths avoided due to increased physical activity)	-1.95 million tons CO_2/year	$8.7 billion total annual savings ($4.94 billion/year savings from improved air quality; $3.8 billion/year savings from increased physical activity)
**Maizlish et al. 2013**	(1) BAU forecasted miles, but with low carbon driving<br>(2) 50% trips <1.5 miles walked, 50% trips 1.5-5 miles biked<br>(3) Optimization of physical activity and emissions reductions acceptable to commuters	(1) 4.4 min/day median active travel time, -21.7 ng/m^3 population-weighted mean $PM_{2.5}$ exposure, traffic injuries<br>(2) 12.4 min/day median active travel time, -46.8 ng/m^3 population-weighted mean $PM_{2.5}$ exposure, traffic injuries<br>(3) 22.0 min/day median active travel time, -106.7 ng/m^3 population-weighted mean $PM_{2.5}$ exposure, traffic injuries	Cardiovascular disease, diabetes, dementia, breast cancer, colon cancer, depression, traffic injuries, deaths, years lived with disability	BAU, 2035	San Francisco Bay Area, California	Not presented	Annually per million population:<br>(2) -3<br>(3) -305	Due to physical activity; injuries:<br>(2) -17,600; 2,456<br>(3) -27,545; 4,524	Due to physical activity; injuries:<br>(2) -1,501; 61<br>(3) -2,404; 113	Compared to 2000 baseline of 27.9 Mton:<br>(1) -33.5%<br>(2) -0.7%<br>(3) -14.5%)	Not presented

**Woodcock et al. 2013**	Three scenarios based on Visions 2030 Walking and Cycling Project:<br>(1) mean of 14.1 minutes walking per day; mean of 6.4 minutes of cycling per day<br>(2) mean of 16.8 minutes walking per day; mean of 9.5 minutes of cycling per day<br>(3) mean of 21.6 minutes walking per day; mean of 18.2 minutes cycling per day	(A) Active travel (MET hours)<br>(B) Exposure to traffic-related injury risk<br>(C) Population weighted exposure to urban transport emissions 1: -0.1 µm $PM_{2.5}$, 2: -0.4 µm $PM_{2.5}$, 3: -0.5 µm $PM_{2.5}$	Ischemic heart disease, stroke, dementia, injuries, diabetes, depression, breast cancer, colon cancer, hypertensive heart disease, lung cancer, respiratory diseases, inflammatory heart disease	actual 2002-2008, accounting year	Urban areas in England and Wales excluding London	Not presented	(A) 1: -3,505 per million per year, 2: -5,129 per million per year, 3: -7,595 per million per year<br>(B) 1: -228 per million per year, 2: -855 per million per year, 3: -867 per million per year<br>(C) 1: -47 per million per year, 2: -137 per million per year, 3: -166 per million per year	Reported in physical activity sensitivity analysis	Reported in physical activity sensitivity analysis	(1) 15.6 Mt (26% reduction)<br>(2) 44.1 Mt (73% reduction)<br>(3) 50.2 Mt (83% reduction)	Not presented
**Macmillan et al. 2014**	(1) Regional cycle network based on 2010 Aukland Regional Council's 30-year transport strategy<br>(2) Arterial segregated bicycle lanes (ASBL)<br>(3) Self-explaining roads (SER)<br>(4) ASBL + SER<br>Scenarios 2-4 related to Aukland's road network compared to international bicycling infrastructure standards	(1) 5% cycling mode share by 2051; cumulative -3.5 billion light vehicle kilometers traveled<br>(2) 20% cycling mode share by 2051; cumulative -7 billion light vehicle kilometers traveled<br>(3) 5% cycling mode share by 2051; cumulative -10 billion light vehicle kilometers traveled<br>(4) 40% cycling mode share by 2051; cumulative -18.5 billion light vehicle kilometers traveled	Traffic injury: serious cyclist injuries, cyclist fatalities, car occupant fatalities<br>Air pollution: mortality, hospitalizations, COPD incidence, restricted activity days<br>All-cause mortality (due to increase in physical activity)	BAU (no investment in cycling); 1991-2051	Aukland, New Zealand	Not presented	Not presented	Not presented	(1) -650 cumulative (tens per year)<br>(2) -1,850 cumulative (hundreds per year)<br>(3) -650 cumulative (tens per year)<br>(4) -4,000 cumulative (hundreds per year)	(1) -3 megatons<br>(2) -8 megatons<br>(3) -13 megatons<br>(4) -26 megatons	Mortality:<br>(1) -2,000,000 NZD<br>(2) -5,700,000 NZD<br>(3) -2,000,000 NZD<br>(4) -4,000,000 NZD<br>Monetization also presented for injuries, fatalities, and air pollution in Table 3
**Xia et al. 2015**	Referencing 30-Year Plan for Greater Adelaide: (1) 5% of vehicle kilometers traveled (VKT) replaced with cycling<br>(2) 10% of VKT replaced with cycling<br>(3) 20% of VKT replaced with public transport<br>(4) 30% of VKT replaced with public transport<br>(5) 10% of VKT replaced with cycling, and 30% of VKT replaced with public transport	(1) -0.13 µg/m^3 (-8.5%) $PM_{2.5}$, 40-70% of population with sufficient physical activity, probability of traveler injury<br>(2) -0.13 µg/m^3 (-8.6%) $PM_{2.5}$, 55-78% of population with sufficient physical activity, probability of traveler injury<br>(3) -0.17 µg/m^3 (-11.5%) $PM_{2.5}$, 35-68% of population with sufficient physical activity, probability of traveler injury<br>(4) -0.33 µg/m^3 (-21.6%) $PM_{2.5}$, 40-72% of population with sufficient physical activity, traffic injury<br>(5) -0.39 µg/m^3 (-26.0%) $PM_{2.5}$, 65-80% of population with sufficient physical activity, traffic injury	PM2.5: cardiovascular disease, respiratory disease, lung cancer; Physical activity: colon cancer, breast cancer, ischemic heart disease, stroke, type 2 diabetes, falls, depression; Traffic: injury	BAU, 2030	Adelaide, Australia	Not presented	Total Annually:<br>(1) -2,113<br>(2) -4,363<br>(3) -1,892<br>(4) -2,948<br>(5) -7,674	Not presented	Total Annually:<br>(1) -160<br>(2) -326<br>(3) -122<br>(4) -187<br>(5) -542	(1) 191,313 tons/year<br>(2) 238,626 tons/year<br>(3) 477,252 tons/year<br>(4) 715,878 tons/year<br>(5) 954,503 tons/year	Not presented; instead measures of "economic wellbeing" presented in "Additional file 1" Table A4c
**Sabel et al. 2016**	Adopted urban climate mitigation policies related to transportation, buildings, and energy	Air Pollution, noise, physical activity (presented in "Additional file 1", Table A2a & A2b)	Mortality, morbidities, DALYs presented in "Additional file 1", Table A3	BAU; 2010, 2020	Kuopio, Finland; Rotterdam, Netherlands; Stuttgart, Germany; Basel, Switzerland; Thessaloniki, Greece; Xi'an, China; Suzhou, China	Various (presented in "Additional file 1", Table A3)	Various (presented in "Additional file 1", Table A3)	Various (presented in "Additional file 1", Table A3)	Various (presented in "Additional file 1", Table A3)	Various (presented in "Additional file 1", Table A3)	Not presented; instead measures of "economic wellbeing" presented in "Additional file 1" Table A4c
**Shindell et al. 2016**	Transportation reductions avoiding 0.03°C warming in 2030 and 0.15°C in 2100 assuming a constant rate of decrease between 2015 and 2030 (surface transport emissions reduction of 75% over baseline)	Reduction in $PM_{2.5}$ of 0.1 to 0.9 µ/m^3	Premature mortality	RCP8.5; 2030, 2050, 2100	US	Not presented	Not presented	Not presented	-14,000 premature deaths/year; -120,000 cumulative deaths 2015-2030	76 mW/m^2 in 2050; 136 mW/m^2 in 2100	US$400B
**Stevenson et al. 2016**	City-specific compact city models with 30% increase in land-use density, 30% reduction in average distance to public transportation, 30% increase in diversity of land use, and 10% modal shift away from private motor vehicles over baseline	24.1% to 72.1% increase in travel-related METs; 3.2% to 12.4% decrease in transport-related particulate emissions	Cardiovascular disease, type 2 diabetes, respiratory disease, road trauma	Existing land use and transport mode choices	Melbourne, AU; Boston, USA; London, UK; São Paulo, Brazil; Copenhagen, Denmark; Delhi, India	Not presented	393 to 826 per 100,000 population	Not presented	Not presented	Not presented	Not presented

Supplementary table 4 – Health co-benefits of mitigation modeling studies - diet

	Scenarios and Policy Relevance	Associated Change in Health Related Exposure	Health Outcomes Estimated	Baseline & Time Period	Study Location	Percent Change in Risk	Change in DALYs	Change in YLLs	Change in Mortality (deaths unless otherwise specified)	per CO2 Equivalent Averted	Monetized Benefits
**Friel et al. 2009**	Agricultural improvements and 30% reduction in livestock production to achieve UK Committee on Climate Change recommendation of 50% reduction over 1990 emissions levels by 2030	30% reduction in livestock consumption associated with 30% reduction in intake of (1) saturated fat and (2) cholesterol	Ischemic heart disease	2010, 2030	UK & São Paulo city	Not presented	(1) -23 to -175 per million per year, -15% to -16%<br>(2) not calculated, data not available	(1) -21 to -165 per million per year, -16% to -17%<br>(2) -9 to -55 per million per year, -5% to -7%	(1) -130 to -290 per million per year, -13% to -17%<br>(2) -40 to -70 per million per year, -4% to -5%	per 9 $MtCO_2e$ in 2030	not presented
**Aston et al. 2012**	Increase in proportion of vegetarians and reduced red and processed meat consumption across the population in support of UK Committee on Climate Change target of 80% reduction over 1990 emissions levels by 2050	42-44% reduction in red and processed meat intake	(A) Coronary heart disease<br>(B) Type 2 Diabetes<br>(C) Colorectal Cancer	2000/2001 (diet), 2004 (disease risk), 2009 (population)	UK	(A) -6.4% to -9.7%<br>(B) -7.5% to -12.0%<br>(C) -7.7% to -12.2%	(A) -50,960 total in 2004<br>(B) -5,421 total in 2004<br>(C) -13,762 total in 2004	Not presented	Not presented	per 27.8 MtCO2e in 2009<br>per 0.45 tonnes CO2e/person/year	not presented
**Scarborough et al. 2012**	Dietary scenarios from 4th carbon budget of UK Committee of Climate Change, which was established by Climate Change Act for advising government on how to meet GHG emissions targets	(1) 40% reduction in milk & eggs, 64% reduction in meat, 60% increase in fruits & vegetables; (2) 75% reduction in red meat, 45% increase in white meat; (3) 50% reduction in white meat, 10% increase in fruits and vegetables	(A) Total averted deaths<br>(B) CHD deaths averted<br>(C) Cancer deaths averted	2008	UK	Not presented	Not presented	Not presented	(A) 1: -11.7% to -20.3%, 2: -0.7% to -1.1%, 3: -2.9% to -4.9%<br>(B) 1: -20.3%, 2: -1.2%, 3: -5.1%<br>(C) 1: -9.6%, 2: -0.3%, 3: -2.5%	(1) per 19% reduction (vs. 2005) in agricultural GHG emissions<br>(2) per 9% reduction (vs. 2005) in agricultural GHG emissions<br>(3) per 3% reduction (vs. 2005)in agricultural GHG emissions	not presented
**Springmann et al. 2016**	No specific policy investigated, but analysis conducted in context of role of diet in contributing to climate and health outcomes and targets globally; reference and scenarios based on global dietary forecast (United Nations Food and Agriculture Organization) and recommendations	(1) 25-190% increase in fruit and vegetable consumption, 56-78% reduction in meat consumption, (2) 39% increase in fruit & vegetable consumption, 100% reduction in meat consumption, (3) 54% increase in fruit & vegetable consumption, 100% reduction in meat; all scenarios reflect target energy intakes based on age and sex composition of population for maintenance healthy body weight	Deaths due to Coronary heart disease, stroke, cancer, type 2 Diabetes	2050	Global by region	Not presented	Not presented	(1) Total: -79M globally<br>(2) Total: -114M globally<br>(3) Total: -129M globally	(1) Total: 5.1M avoided deaths globally, approx. 0.6 per thousand<br>(2) Total: 7.3M avoided deaths globally, approx. 0.8 per thousand<br>(3) Total: 8.1M avoided deaths globally, approx. 0.95 per thousand	(1) reduction of (11.4 - 8.1) Gt/yr in 2050 food-related GHG emissions<br>(2) reduction of (11.4 - 4.2) Gt/yr in 2050 food-related GHG emissions<br>(3) reduction of (11.4 - 3.4) Gt/yr in 2050 food-related GHG emissions	Economic benefits of 1-31 trillion US dollars, 0.4-15% of global GDP in 2050
**Springmann et al. 2017**	GHG emissions price of \$52/tCO_2e applied to food commodities relevant to each scenario:<br>(1) All food commodities<br>(2) Exclude fruit, vegetables, staples and legumes<br>(3) Animal-based foods (meats, eggs, milk)<br>(4) Red meat (beef, lamb, pork)<br>(5) Beef<br>(6) Regionally optimized tax scenarios for greatest health benefit<br>+ additional scenario variants: compensation for income losses; fruit and vegetable subsidies	Prevalence of overweight and obesity from weight distributions based on food availability and mean BMI;	Coronary heart disease, stroke, type 2 diabetes, site-specific cancers, all other disease risk	2020	Global by region	Not presented	Not presented	Not presented	(1) 145,790 avoided deaths globally<br>(2) 151,160 avoided deaths globally<br>(3) 136,550 avoided deaths globally<br>(4) 144,570 avoided deaths globally<br>(5) 91,420 avoided deaths globally<br>(6) 509,480 avoided deaths globally	(1) 1.0 $GtCO_2e$<br>(6) 919 $MtCO_2e$	Not presented
**Hallström et al. 2017**	Reductions in GHG associated with hypothetical healthier alternative diets in the US presented as percentage of US Climate Action Plan targets	(1) 45% reduction in red and processed meat, 101% increase in fruits and vegetables; (2) 73% reduction in red and processed meat, 111% increase in fruits and vegetables, (3) 100% reduction in red and processed meat, 121% increase in fruits and vegetables	(A) Coronary heart disease<br>(B) Type 2 Diabetes<br>(C) Colorectal Cancer	2013	US	(A) 1: -40%, 2: -45%, 3: -45%<br>(B) 1: -35%, 2: -41%, 3: -43%<br>(C) 1: -20%, 2: -25% 3: -29%	Not presented	Not presented	Not presented	(1) 68 kg CO2/capita/year (6% of President's Climate Action Plan target of 17% reduction below 2005 by 2020)<br>(2) 306 kg CO2/capita/year (15% of target)<br>(3) 543 kg C02/capita/year (23% of target)	Health care cost savings of US \$77B-\$93B per year
